# Fibrotic scarring prevents optic nerve regeneration despite preserved axonal growth potential in adult killifish

**DOI:** 10.3389/fnins.2026.1745022

**Published:** 2026-02-18

**Authors:** Julie D. De Schutter, Luca Masin, Anyi Zhang, Lieve Moons, Steven Bergmans

**Affiliations:** Division of Animal Physiology and Neurobiology, Department of Biology, Neural Circuit Development and Regeneration Research Group, Leuven Brain Institute, KU Leuven, Leuven, Belgium

**Keywords:** retinal ganglion cell, axonal regeneration, scarring, axon misguidance, inflammation, fibrosis, killifish, optic nerve transection

## Abstract

Adult mammals exhibit limited regenerative capacity in the central nervous system (CNS), leading to irreversible deficits following injury or disease. Effective strategies to restore CNS function remain lacking. For retinal disorders, whole-eye transplantation has emerged as a promising approach, yet reinnervation of visual brain targets remains a major challenge. Here, we evaluated the killifish—a teleost fish species displaying robust regenerative capacities during young adulthood and mammalian-like regenerative traits at old age—as a translational model for whole-eye transplantation. We analyzed axonal regeneration following complete optic nerve transection (cONT), an injury paradigm relevant to whole-eye transplantation, in both young adult and aged individuals. Unexpectedly, retinal ganglion cells (RGCs) in adult killifish failed to reinnervate their brain target after cONT, in contrast to regeneration-competent zebrafish. Despite this failure, RGCs retained high intrinsic growth potential, evidenced by aberrant axonal projections within the retina. The inability to reestablish brain connectivity, combined with inflammation and intrinsic vulnerability, likely underlies the severe RGC loss (~75%) in both age groups. We identified the formation of a dense, collagen-rich gliofibrotic scar at the lesion site as a major barrier to axonal regeneration. Intriguingly, partial optic nerve transection, which markedly reduced scar formation, improved RGC survival, facilitated robust axonal regeneration and restored target reinnervation. Together, these findings establish the killifish as a powerful model to study scar-mediated inhibition of CNS regeneration, with important implications for advancing CNS repair strategies, including whole-eye transplantation.

## 1 Introduction

Globally, visual impairment affects more than one billion people, primarily due to structural or pathological disruptions in the cornea, lens, or retina (Steinmetz et al., 2021; World Health Organization, 2023). While disorders of the cornea and lens—such as refractive errors and cataracts—can be effectively managed with corrective lenses or surgical intervention, retinal pathologies such as glaucoma (~80 million cases), diabetic retinopathy (~103 million cases), and age-related macular degeneration (~200 million cases) remain major causes of irreversible blindness worldwide (Teo et al., 2021; Glaucoma Research Foundation, 2022; Furtado et al., 2024). This stems from the limited regenerative capacity of the human central nervous system (CNS), which includes retinal neurons. A radical strategy now under exploration is whole-eye transplantation from deceased donors, including their optic nerves. The Total Human Eye Allograft (THEA) initiative, recently achieved the first human eye transplant with successful anatomical integration and no immune rejection, though visual recovery has yet to be attained (Ceradini et al., 2024).

In contrast to mammals, teleost fish such as zebrafish and goldfish display an exceptional capacity for tissue regeneration, including within the CNS (McQuarrie, 1985; Kato et al., 1999; Diekmann et al., 2015). Following optic nerve crush (ONC), both species demonstrate functional visual recovery within 2–3 weeks, characterized by nearly complete retinal ganglion cell (RGC) survival, robust axonal regeneration, and full target reinnervation (Edwards et al., 1981; Murray and Edwards, 1982; McQuarrie, 1985; Diekmann et al., 2015; Van Houcke et al., 2017; Ferreira, 2024; De Schutter et al., 2025). More recently, the African turquoise killifish has emerged as a valuable complementary model for studying axonal regeneration. This naturally short-lived teleost (~24–28 weeks lifespan) undergoes rapid aging and displays conserved molecular and cellular hallmarks of human aging (Kim et al., 2016; Vanhunsel et al., 2021; Bergmans et al., 2023a, 2024; de Bakker and Valenzano, 2023). Notably, its regenerative capacity declines with age: while young adult killifish regenerate RGC axons efficiently post-ONC and regain vision, aged individuals fail to recover functionally, showing incomplete target reinnervation, scarring and increased RGC loss (Vanhunsel et al., 2022a, 2022b; De Schutter et al., 2025). These features make killifish a compelling model for investigating age-associated declines in neurorepair.

Although the ONC injury paradigm is widely used to study CNS axonal regeneration, it does not recapitulate the full extent of injury associated with whole-eye transplantation, in which the optic nerve is completely severed. Optic nerve transection (ONT) therefore represents a more clinically relevant model in this context, as successful regeneration must occur across a complete disconnection of the ocular compartment from the brain. Indeed, in contrast to ONC, ONT involves transection of both the optic nerve perineurium and the axonal shafts. Remarkably, both zebrafish and goldfish retain the ability to fully reinnervate central visual targets and restore visual function following ONT (Meyer et al., 1985; Zou et al., 2013; Shen et al., 2025), underscoring the robustness of their regenerative potential.

How killifish respond to ONT has remained unexplored. Here, we address this gap and reveal that, neither young adult (6-week-old) nor aged (18-week-old) killifish are capable of reinnervating the brain after complete ONT (cONT) and both exhibit severe RGC loss. Intriguingly, intrinsic axonal growth capacity is preserved, as evidenced by regenerating axons accumulating at the retinal margin. The failure to reinnervate visual targets is most likely due to the formation of a dense collagen-rich fibrotic scar at the transection site, bordered by astrocyte-like cells. Strikingly, when only a partial optic nerve transection (pONT) is performed in young adult killifish, scarring is minimal and axons successfully reinnervate central targets. These findings establish the killifish as a powerful model to dissect scar-associated mechanisms underlying regeneration failure and to explore strategies for promoting CNS repair, particularly relevant in the context of whole-eye transplantation.

## 2 Materials and methods

### 2.1 Animal housing

#### 2.1.1 Killifish

Killifish (*Nothobranchius furzeri*) of the GRZ-AD inbred strain were bred and housed as described (Vanhunsel et al., 2021; Bergmans et al., 2023b). Briefly, fish were maintained under standardized conditions, i.e., a 12 h/12 h light–dark cycle, pH 7, and a conductivity of 600 μS, in a recirculating aquatic system (ZebTec system, Techniplast), warranting a constant water temperature of 28 °C. Adult fish (4 weeks and older) were kept in a 3.5 L tank at a density of three females and one male, and fed twice a day with fresh frozen *Chironomidae* species (Ocean nutrition). As male and female killifish differ in their growth and aging pattern (Reichard et al., 2022), exclusively young adult (6-week-old) and aged (18-week-old) female killifish of the GRZ-AD inbred strain were used to exclude potential sex-based growth effects from the datasets. The two age groups were selected based on the survival curve (Van Houcke et al., 2021) and previously published work on optic nerve injury (Vanhunsel et al., 2022a; De Schutter et al., 2025). All experiments were approved by the institutional Animal Ethics Committee of the KU Leuven, strictly following the European Communities Council Directive of 20 October 2010 (2010/63/EU).

#### 2.1.2 Zebrafish

Zebrafish (*Danio rerio*) of the *Tg(Tru.gap43:eGFP)* line were raised and maintained under standard laboratory conditions as described (Van Dyck et al., 2023). Briefly, fish were housed in a recirculating aquatic system (ZebTec, Techniplast) with a temperature of 28 °C, a conductivity of 650 μS, pH of 7.4, on a 14 h/10 h light/dark cycle. Zebrafish were fed twice a day with both dry food and brine shrimp (*Artemia Salina nauplii*, Ocean nutrition). All experiments were conducted on 20-week-old adult zebrafish, including both males and females. All experiments were approved by the institutional Animal Ethics Committee of the KU Leuven, strictly following the European Communities Council Directive of 20 October 2010 (2010/63/EU).

### 2.2 Optic nerve transection models

#### 2.2.1 Complete optic nerve transection (cONT)

Unilateral cONT was performed as an adaptation of the previously described ONC protocol (Vanhunsel et al., 2022a, 2023; Beckers et al., 2023). Briefly, fish were anaesthetized by submersion in 0.03% tris-buffered-tricaine (MS-222, Sigma) in system water and placed under a stereo microscope. The dermal layer of the cornea of the left eye was incised and the eyeball was gently lifted from its orbit. Dorsal ocular muscles were transected to expose the optic nerve and ophthalmic artery. The optic nerve was fully transected at a distance of 500 μm from the optic nerve head. Fish presenting with arterial damage, observed by bleeding, were euthanized by submersion in 0.1% tris-buffered-tricaine and excluded from the experiment. The eye was repositioned in its socket, and the fish were reawakened in a recovery tank containing system water. Optic nerve regeneration was evaluated at multiple days post-injury (dpi).

#### 2.2.2 Partial optic nerve transection (pONT)

Fish were anesthetized and the optic nerve was exposed as described for a cONT. A unilateral partial transection was then performed 500 μm from the optic nerve head, cutting approximately 50% of the nerve from the dorsonasal side. Fish with ophthalmic artery damage or with a near complete nerve transection—defined as a lesion exceeding two-thirds of the optic nerve diameter—were euthanized and excluded from further analyses. The eye was repositioned in its socket and the fish were reawakened as described above. Axonal regeneration was studied at different timepoints post-injury.

### 2.3 Tracing methods

Tectal reinnervation and RGC regeneration in the retina were evaluated using anterograde and retrograde tracing, respectively, adapted from previously published protocols (Vanhunsel et al., 2022a, 2023). Similarly to the tracing protocol post-ONC, fish were anesthetized using 0.03% tris-buffered-tricaine and placed under a stereo microscope. The dermal layer of the cornea was cut and the eye lifted from its socket. The connective tissue surrounding both the proximal and distal segments of the optic nerve was meticulously cleared and a biocytin wad was placed at the transection site, touching both the proximal (for retrograde tracing) and distal end (for anterograde tracing) of the nerve. Fish were awakened for 3 h to allow for tracer transport, saturating the regenerating RGC somas and axons with biocytin and subsequently euthanized prior to transcardial perfusion and tissue collection.

### 2.4 Tissue collection

To assess the regeneration process in young adult and aged killifish (6- and 18-week-old, respectively) after ONT, fish were sacrificed at defined post-injury timepoints. Euthanasia was performed by an overdose of tris-buffered-tricaine (0.1% in system water), followed by transcardial perfusion with phosphate-buffered-saline (PBS) and 4% paraformaldehyde (PFA) as described previously (Bergmans et al., 2023c). Eyes, brains, or complete visual systems were collected and post-fixed in 4% PFA (1 h for eyes and brains, overnight for visual systems), then rinsed three times in PBS and stored in storage buffer (0.4 M NaN3 in PBS) at 4 °C until further use. Complete visual systems, comprising the eyes, optic projections, and brain, were dissected as a single unit to maintain the structural integrity of the optic nerves. For whole-mount preparations, eyes were further dissected to isolate the retina (referred to as whole-mounts, WMs), which were fixed for an additional hour in 4% PFA before being stored in storage buffer at 4 °C. Tissues intended for cryosectioning were cryo-protected in a graded sucrose series (10, 20, and 30%) before embedding. Eyes, brains, and complete visual systems were serially sectioned using a Cryostat NX70 (Epredia) along the sagittal, coronal, and horizontal planes, respectively, and stored at −20 °C.

### 2.5 Tissue processing

#### 2.5.1 Biocytin visualization and immunohistochemistry

Tectal reinnervation was visualized by biocytin-labeling of anterogradely traced brain sections. Briefly, brain cryosections were defrosted, dried at 37 °C, and rehydrated using distilled water. Sections were permeabilized by three rinses for 5 min in 0.1% Triton X-100 in PBS (0.1% PBST). Next, biocytin was visualized by using an Alexa-coupled streptavidin (1,200 in 0.1% PBST, ThermoFisher) for 2 h, followed by a nuclear stain using 4′,6-diamidino-2-phenylindole (DAPI, 1:1000 in PBS) for 30 min. Slides were mounted with anti-fading mounting reagent Mowiol^®^ (10%, Sigma-Aldrich). Mosaic images of coronal brain sections were acquired using a wide-field epifluorescent microscope (Leica DM6) containing a HC PL FLUOTAR L20X/0.40 CORR objective with a DFC365FX camera.

Regenerating and surviving RGCs were visualized on retinal WMs using biocytin labeling and immunohistochemical staining for RNA Binding Protein with Multiple Splicing 2 (Rbpms2) and acetylated tubulin (aTub). Briefly, WMs were washed twice in 0.5% PBST and permeabilized by a 15 min freeze–thaw step at −80 °C. WMs were blocked for 2 h using pre-immune donkey serum (PID, 1:5 in 2% PBST) prior to overnight incubation with the anti-Rbpms2 and anti-aTub antibodies diluted in 2% PBST with 10% PID (details in Table 1). Tissues were rinsed three times for 5 min with 0.5% PBST before a 2 h incubation with the secondary antibody (Alexa-conjugated donkey anti-primary antibody IgG, 1:200 in 2% PBST with 10% PID, ThermoFisher). WMs were rinsed three times with PBS, followed by another 2 h incubation with an Alexa-coupled streptavidin to visualize biocytin, and with DAPI for nuclear labeling (1,200 and 1:1000, respectively, in PBS). WMs were mounted with anti-fading mounting reagent Mowiol^®^ (10%, Sigma-Aldrich). For quantitative analyses, mosaic images of complete retinal WMs were acquired as described for brain sections above, while representative micrographs were taken using a Zeiss LSM900 with Airyscan 2 confocal microscope with a Plan-Apochromat 20X/0.8 M27 objective.

**Table 1:** List with primary antibodies used for immunohistochemistry.

Antibody	Manufacturer	Catalog number	Host species	Dilution
Anti-aTub	Sigma-Aldrich	T6793	Mouse	1:200
Anti-Blbp	Abcam	Ab110099	Goat	1:500
Anti-Gfap	DAKO	Z0334	Rabbit	1:200
Anti-Lcp1	Custom	NA	Guinea Pig	1:1000
Anti-Rbpms2	Abcam	Ab181098	Rabbit	1:200
Anti-vimentin	Sigma-Aldrich	V5255	Mouse	1:400

Retinal and visual system cryosections were stained either for Brain Lipid-Binding Protein (Blbp), Glial Fibrillary Acidic Protein (Gfap), vimentin, Lymphocyte Cytosolic Protein 1 (Lcp1) and/or biocytin. Briefly, cryosections were defrosted, dried at 37 °C and rehydrated using distilled water. Slides were permeabilized as described for brain sections. Retinal sections stained for vimentin and Lcp1 underwent a heat-mediated acidic antigen retrieval using citrate buffer (pH6.4) at 95 °C for 20 min in a PT module (ThermoFisher). A softer antigen retrieval was performed on visual system cryosections stained for Lcp1; slides were submerged in pre-heated (95 °C) citrate buffer for 20 min at room temperature. After antigen retrieval, slides were washed three times using 0.1% PBST. Aspecific binding of antibodies was blocked by slide submersion in PID (1:5 in 0.1% PBST). Slides were incubated overnight with primary antibodies anti-Blbp, anti-Gfap, anti-vimentin and/or anti-Lcp1 diluted in 1% PBST with 10% PID (details in Table 1). The next day, unbound primary antibody was washed away and the slides were incubated for 2 h with secondary antibodies (Alexa-conjugated donkey anti-primary antibody IgG, 1:200 in 2% PBST with 10% PID, ThermoFisher), followed by 30 min DAPI (1:1000 in PBS), and mounted using Mowiol^®^. Complete retinal and optic nerve mosaics were acquired as described for brain sections. High-magnification images were collected using a Zeiss LSM900 with Airyscan 2 confocal microscope with a Plan-Apochromat 63X/1.4 oil DIC M27 objective.

#### 2.5.2 Histochemistry

Optic nerve morphology and scar formation were visualized with hematoxylin and eosin (H&E) staining. Cryosections of complete visual systems were defrosted, dried at 37 °C, and rehydrated in distilled water. The slides were submerged in Hematoxylin solution (prepared according to Mayer, Sigma-Aldrich) for 3 min and rinsed with running tap water for 10 min. Next, a 10 s stain in Eosin (0.5%, Sigma-Aldrich) was performed followed by two rinses in distilled water and a dehydration series (rinses in 50, 70, and 90% EtOH, two 3 min washes in 100% EtOH, and two 5 min washes in xylene). The slides were mounted with DePex (Sigma-Aldrich) and bright-field imaging was done with a wide-field epifluorescent microscope (Leica DM6) containing a HC PL FLUOTAR L20X/0.40 CORR objective with a DFC365FX camera.

Sirius red staining was performed on complete visual system cryosections to identify collagen deposition in the optic nerve scar after transection (adapted from Vanhunsel et al., 2022a). Briefly, after defrosting and drying the slides at 37 °C, they were placed for 3 min in 70% EtOH and then in distilled water. Next, the slides were incubated for 1 h 30 in Picro-Sirius red solution (1:1000 Direct Red 80, Sigma-Aldrich in 1.3% picric acid in distilled water, Sigma-Aldrich) in the dark. To rinse off the excess Sirius red staining solution the slides were washed 2 times for 5 min with acidified water (1:200 glacial acetic acid, Sigma-Aldrich in distilled water). Next, the slides were dehydrated with 100% EtOH and xylene, each for 5 min, and finally mounted with DePex (Sigma-Aldrich). Pictures of the visual systems were taken under bright-field and polarized light with a wide-field epifluorescent microscope (Leica DM6) containing a HC PL FLUOTAR L20X/0.40 CORR objective with a DFC365FX and DMC2900 camera, respectively.

#### 2.5.3 *In situ* hybridization

To identify the cellular population within the optic nerve scar *in situ* hybridization chain reaction (HCR) V3.0 was performed as previously described (Choi et al., 2018; Van Houcke et al., 2021; Elagoz et al., 2022; Bergmans et al., 2024). Probes were designed using easy_hcr tool^1^ and purchased on Integrated DNA Technologies (IDT). Briefly, cryosections of the visual system were dried at 37 °C, rehydrated in autoclaved PBS-diethyl pyrocarbonate (PBS-DEPC, 0.1% v/v, Acros), and permeabilized with PBS-DEPC-tween (1% Tween 20 in PBS-DEPC) for 10 min. Proteinase K treatment (1:3000 of a 18 mg/mL solution, Sigma-Aldrich, in PBS-DEPC) was performed for 10 min at 37 °C and stopped with 4% PFA for 10 min. Next, sections were pre-hybridized in hybridization buffer (30% formamide; 25% 20X SSC^®^, ThermoFisher; 9 mM citric acid pH 6.0; 0.1% Tween 20; 50 μg/mL Heparin; 2% 50x Denhardt’s solution; 10% Dextran) for 30 min at 37 °C, incubated overnight at 37 °C with probe solution (probes in hybridization buffer, optimized for each probe as detailed in Table 2). Non-hybridized probes were washed away and sections were pre-amplified with amplification buffer for at least 30 min at room temperature (25% 20X SSC^®^; 0.1% Tween 20; 10% Dextran). Hairpin amplifiers (H1 and H2, 3 pmol each) were activated at 95 °C for 1.5 min, cooled 30 min at room temperature, and applied in amplification buffer (1:50, final concentration 40 nM) overnight at room temperature. Nuclei were stained with DAPI (1:1000 in PBS) and slides were mounted with Mowiol^®^ (10%, Sigma-Aldrich). Sections were imaged on a Zeiss LSM900 with Airyscan 2 confocal microscope with a Plan-Apochromat 20X/0.8 M27 objective. Mosaic Z-stacks covering the whole thickness of the section were acquired and visualized as maximum projections.

**Table 2:** List of genes used to design probes for *in situ* hybridization chain reaction.

Gene	Predominant cell type	Number of probe pairs	Final probe pair concentration	Gene ID	RefSeq accession code
*apoeb*	Microglia	20	6.67 nM	LOC107379395	XM_015950130
*cx43*	Astroglia	21	13.34 nM	LOC107395986	XM_015975478
*col1a1a*	Fibroblast	28	20.01 nM	LOC107391613	XM_015968981
*fn1b*	Fibroblast	33	20.01 nM	LOC107381665	XM_015953456
*lum*	Fibroblast	28	20.01 nM	LOC107385817	XM_015959945

### 2.6 Analyses

#### 2.6.1 Retinal ganglion cell (RGC) survival analysis

RGC counts, densities, and retinal surface areas were quantified on retinal WMs using the recently published RGCode2 analysis pipeline (De Schutter et al., 2025). Retinal segmentation was performed with the original RGCode model, which has previously demonstrated robust performance (Masin et al., 2021; De Schutter et al., 2025). For each fish, RGC density was calculated as the total number of detected Rbpms2-positive RGCs divided by the corresponding total retinal area. For experiments involving pONT, retinal quadrants were analyzed independently, with a focus on the dorsonasal and ventrotemporal regions, corresponding to injured and uninjured areas of the optic nerve, respectively, to assess regional differences.

#### 2.6.2 Microglia/macrophage density quantification

Lcp1-positive microglia/macrophages were manually counted on six midsagittal retinal sections per fish, evenly distributed across nasal and temporal regions, in both young adult and aged killifish. For each section, two regions of interest (ROIs) were defined in the dorsal and ventral retina. ROIs were positioned at a distance of ~100 μm from the optic nerve head and extended over a length of ~300 μm along the retinal radius. Each ROI spanned the full retinal thickness, ranging from the ganglion cell layer to the outer nuclear layer, while photoreceptor inner and outer segments were excluded due to high autofluorescence. Cell densities (cells/mm^2^) were calculated as the number of Lcp1-positive cells per ROI area. Next, per-fish average densities were calculated from the 12 analyzed ROIs per fish, and, when applicable, normalized to corresponding uninjured controls to assess injury-induced immune response. These per-fish averages were used for statistical analysis and graph plotting.

#### 2.6.3 Statistical analysis

All analyses were conducted on original, non-saturated micrographs. For figure display, linear contrast adjustments were applied uniformly across comparable images. Statistical tests included ANOVA, t-tests, and U-tests as specified in the figure legends. When datasets did not meet normality criteria according to the Shapiro–Wilk test, medians were compared using the Kruskal–Wallis and Mann–Whitney U-tests; otherwise, means were analyzed using Welch’s ANOVA. A *p*-value of ≤ 0.05 was considered statistically significant. Statistical analyses were performed either with the Python library *statsmodels* or GraphPad Prism V10.0.0, while graphing was done using Prism. Sample sizes are reported in the respective figure legends.

## 3 Results

### 3.1 Failed axonal regeneration after complete optic nerve transection (cONT) in young adult and aged killifish

**Figure 1:**
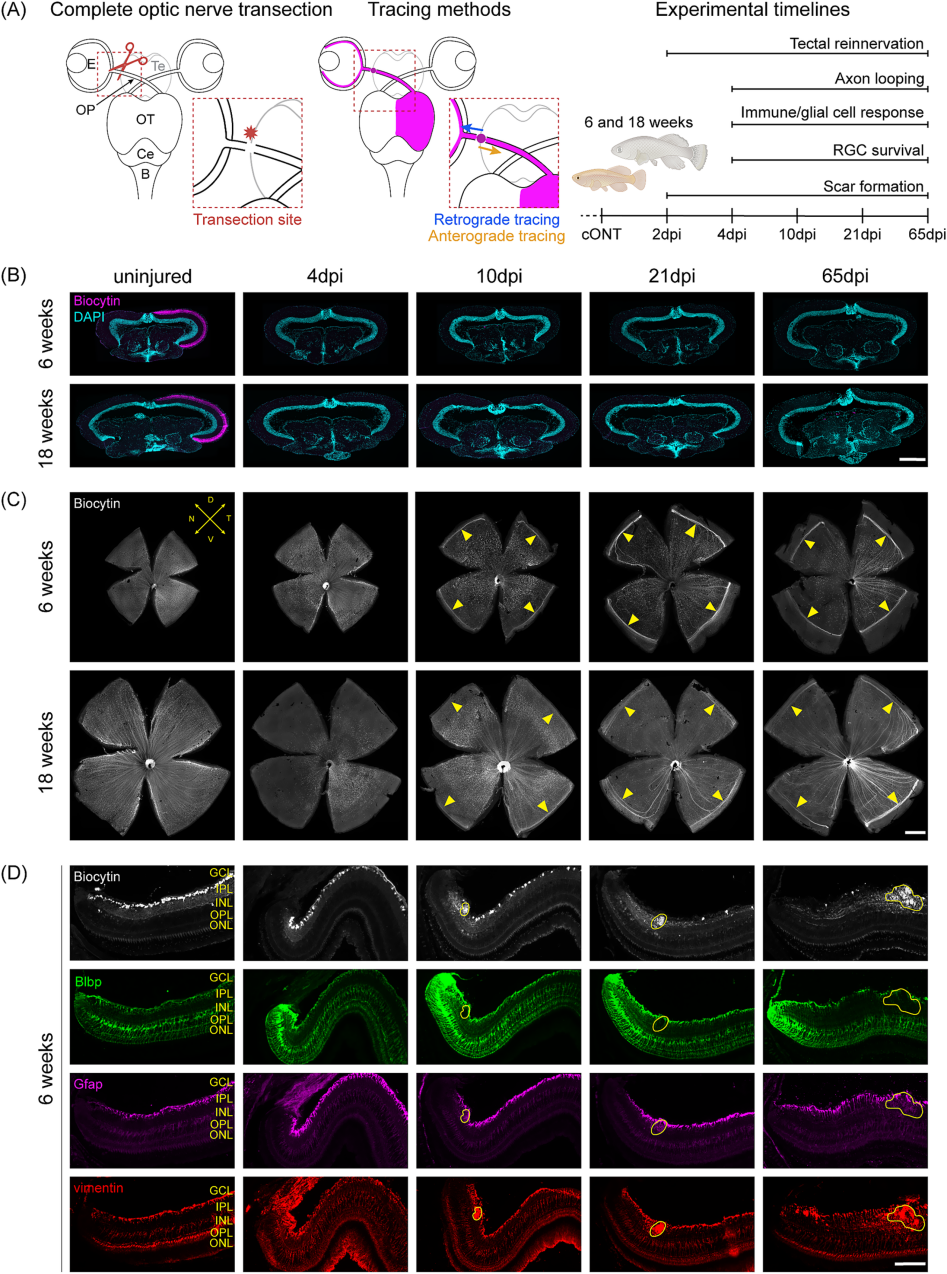
Failed tectal reinnervation and aberrant axonal growth after complete optic nerve transection (cONT). **(A)** Schematic overview of the cONT injury paradigm (dorsal view of the visual system), tracing approaches (retrograde and anterograde biocytin labeling), and experimental timelines used to assess axonal regeneration in young adult (6-week-old) and aged (18-week-old) killifish. With assets from Biorender.com. **(B)** Representative biocytin-labeled coronal optic tectum sections from anterogradely traced young adult and aged killifish (*N* = 6 per condition). In the uninjured condition, biocytin-positive RGC axons (magenta) innervate the target layers in the optic tectum, whereas no tectal reinnervation is observed following cONT up to 65 dpi. Scale bar = 200 μm. **(C)** Representative biocytin-stained retinal WMs from retrogradely traced young adult and aged killifish (*N* ≥ 5 per condition) showing aberrant RGC axon looping at the retinal margin from 10 dpi onwards (yellow arrowheads). The axon loops become more pronounced over time in both age groups. Scale bar = 500 μm. **(D)** Representative micrographs of peripheral retinal sections from young adult killifish after cONT stained for biocytin, Blbp, Gfap, and vimentin (*N* = 6 per condition). From 10 dpi onward, aberrant looping of biocytin-positive RGC axons (yellow circle) is observed beneath the GCL within the IPL. This looping occurs in a Blbp-negative region that shows increased Gfap and vimentin immunoreactivity, indicative of a reactive glial scaffold. The extent of this region of aberrant axonal growth increases over time. Scale bar = 100 μm. B, Brainstem; Blbp, Brain Lipid-Binding Protein; Ce, Cerebellum; cONT, complete optic nerve transection; DAPI, 4′,6-diamidino-2-phenylindole; D, dorsal; dpi, days post injury; E, Eye; GCL, ganglion cell layer; Gfap, Glial Fibrillary Acidic Protein; INL, inner nuclear layer; IPL, inner plexiform layer; N, nasal; ONL, outer nuclear layer; OP, optic projections; OPL, outer plexiform layer; OT, optic tectum; RGC, retinal ganglion cell; T, temporal; Te, telencephalon; V, ventral, WM, whole-mount.

Previous studies using ONC have shown that young adult killifish exhibit robust axonal regeneration, with full reinnervation of their tectal targets, whereas aged fish display only limited brain reinnervation (Vanhunsel et al., 2022a). To evaluate regeneration following cONT, young adult (6-week-old) and aged (18-week-old) female killifish were subjected to anterograde tracing and sacrificed at multiple timepoints post-injury (Figure 1A). Remarkably, no evidence of brain reinnervation was observed in either age group over 65 days following cONT (Figure 1B).

Given the high intrinsic axonal growth potential of young adult killifish (Vanhunsel et al., 2022a), we examined retinal WMs using retrograde biocytin tracing to detect aberrant axonal growth. This method allows visualization of all RGC axons that have regrown at least up to the injury site, where the tracer-soaked wad is applied during retrograde tracing (Figure 1A; Supplementary Figure S1A). Ectopic RGC axons were observed looping at the retinal margin as early as 10 dpi in both age groups (Figure 1C, yellow arrowheads). The looping phenotype arises as regenerating RGC axons encounter the injury site (where they take up biocytin during tracing), turn back toward the eye, and aberrantly extend within the retina (Supplementary Figure S1A). Over time, an increase in the width of the aberrant axonal loops was observed in both age groups (Figures 1C,D), suggesting repeated looping of regenerating RGC axons. However, compared to young adults, aged 18-week-old killifish exhibit a lower axonal density in the region of looping at all timepoints after cONT, likely reflecting the reduced intrinsic growth potential of aged killifish RGCs, as previously reported after ONC (Vanhunsel et al., 2022a). To distinguish regenerating axons, visualized via retrograde biocytin tracing, from those sprouting of newborn RGCs, we performed immunostaining for aTub to label all axons (Supplementary Figure S1B). Zoomed images reveal that the co-labeled biocytin- and aTub-positive regenerating axons, that loop within the retina, are flanked by biocytin-negative, aTub-positive axons positioned more peripherally—likely originating from newborn RGCs and most visible at 21 and 65 dpi (Supplementary Figure S1B). The occurrence of this axonal sprouting is less pronounced in aged fish, consistent with their reduced retinal neurogenic capacity at old age (Vanhunsel et al., 2021; Bergmans et al., 2023b). Furthermore, in both young adult and aged killifish, biocytin-positive RGC somas are clearly visible in the more central retina (Supplementary Figure S1B). Due to peripheral retinal addition of newborn neurons in teleosts, these central RGCs represent older neurons (Bergmans et al., 2023b), supporting their identification as regenerating RGC axons.

While the RGC axons extended from the optic nerve head to the retinal margin via the nerve fiber layer, retinal cross-sectioning revealed that the axonal looping was localized to the inner plexiform layer, immediately beneath the ganglion cell layer, in a spatially restricted pattern (Figure 1D, yellow lining). Initially adjacent to the retinal margin (10 dpi), looping axons appeared more centrally over time (Figures 1C,D), due to the continuous addition of neurons at the retinal periphery by the ciliary marginal zone (Bergmans et al., 2023b). Immunostaining for glial markers further revealed that regions containing axonal loops within the retina lacked Blbp expression (Figure 1D, yellow lining), a Müller glia–specific radial glial marker, indicating the absence of typical Müller glial radial fibers in the region of looping. Instead, these areas showed enrichment of Gfap- and vimentin-signal, likely representing astrocyte-like cell processes and/or Blbp-negative Müller glial protrusions. High-magnification images of retinal sections co-stained with Gfap and biocytin further confirm the close contact between the Gfap-positive glial processes and the biocytin-positive looping axons (Supplementary Figure S1C). This association is particularly prominent during early axonal looping (10 and 21 dpi), as evidenced by overlapping biocytin and Gfap signals. In all, this suggests that (astro)glial scaffolds may contribute to guiding/facilitating intra-retinal axon looping.

In summary, RGCs in both young adult and aged killifish fail to reinnervate their brain targets following cONT, yet retain the capacity to regrow axons, forming aberrant loops at the retinal margin within a glial scaffold.

### 3.2 Severe RGC loss and differential immune response after cONT in young adult and aged killifish

**Figure 2:**
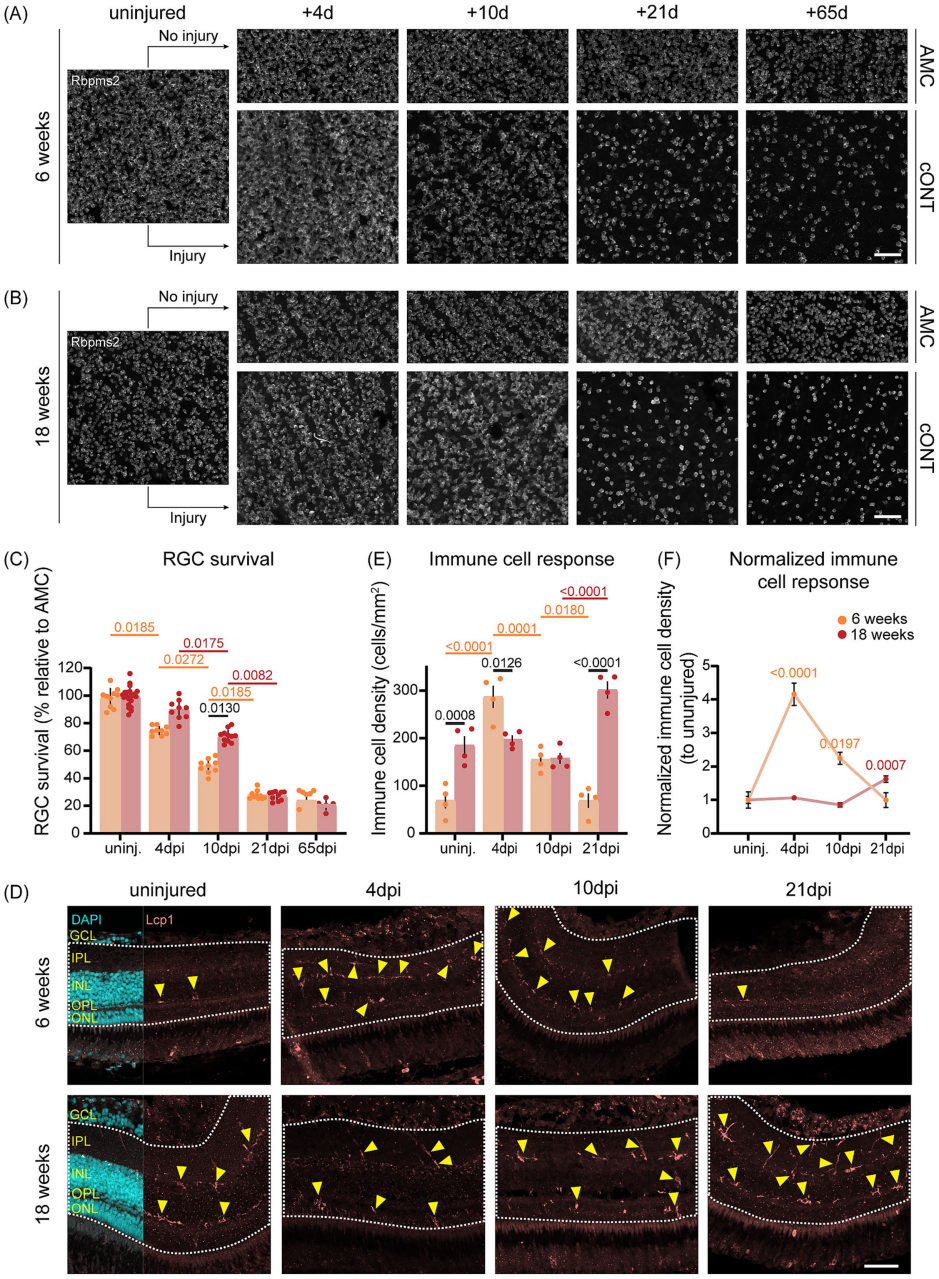
Age-dependent difference in retinal ganglion cell survival and immune response after cONT. **(A,B)** Representative Rbpms2-stained micrographs of retinal whole-mounts from young adult [6-week-old, **(A)**] and aged [18-week-old, **(B)**] killifish (*N* ≥ 5 per condition) with and without injury (cONT). Both age groups demonstrate marked RGC loss at 21 and 65 dpi compared to their uninjured AMCs, retaining similar low RGC densities after cONT. At 10 dpi, aged killifish display visibly higher RGC densities than young adults. Scale bar = 50 μm. **(C)** Quantification of RGC survival in adult killifish WMs after cONT reveals progressive loss in young adults starting from 4 dpi, while old fish show a milder decline during the first 10 dpi. By 65 dpi, both groups converge to ~25% RGC survival. Statistical analysis: two-way Kruskal–Wallis ANOVA and *post hoc* Mann–Whitney *U* tests with Bonferroni correction (*N* ≥ 5 per condition, mean ± SEM). Orange, red, and black *p*-values indicate differences between timepoints in young adults, aged fish, and between age groups at specific timepoints, respectively. **(D)** Representative Lcp1-stained (pink) midsagittal retinal sections from young adult and aged killifish after cONT (*N* = 4 per condition). Old killifish display a higher microglia/macrophage density in the uninjured retina compared to their younger counterparts. Young adults show a peak in immune activation at 4 dpi, whereas aged fish exhibit a delayed neuroinflammatory response only peaking at 21 dpi. The analyzed region of interest is delineated by a white dotted line, and individual microglia/macrophages are indicated by yellow arrowheads. Scale bar = 50 μm. **(E)** Quantification of retinal microglia/macrophage density showing higher baseline levels in aged compared to young adult killifish. Following cONT, immune cell density in young adult fish rapidly increases, peaking at 4 dpi, and surpasses that of aged fish. By 21 dpi, densities return to baseline in young adults, whereas aged fish show maximal accumulation. Statistical analysis as in **(C)** (*N* = 4 per condition, mean ± SEM). Orange, red, and black *p*-values correspond to intra- and inter-age group comparisons as indicated in **(C)**. **(F)** Normalized retinal microglia/macrophage densities relative to uninjured AMC. In young adults, immune activation peaks at 4 dpi with a fourfold increase, while aged fish reach maximal densities at 21 dpi, nearly doubling relative to baseline. Statistical analysis: ordinary one-way ANOVA and *post-hoc* Turkey’s multiple comparison test (*N* = 4 per condition, mean ± SEM). Orange and red *p*-values indicate intra-age comparisons to the uninjured condition for young and aged fish, respectively. AMC, age-matched controls; cONT, complete optic nerve transection; d, days; DAPI, 4′,6-diamidino-2-phenylindole; dpi, days post injury; GCL, ganglion cell layer; IPL, inner plexiform layer; INL, inner nuclear layer; Lcp1, lymphocyte cytosolic protein 1; OPL, outer plexiform layer; ONL, outer nuclear layer; Rbpms2, RNA binding protein with multiple splicing 2; RGC, retinal ganglion cell; SEM, standard error of the mean; Uninj., uninjured.

Since recent studies have demonstrated that killifish RGCs are more susceptible to injury than those of zebrafish (De Schutter et al., 2025), and that some RGC subtypes can survive axotomy without re-establishing functional synapses post-ONC (Vanhunsel et al., 2022a), we assessed whether ONT results in extensive RGC loss. RGC survival was studied by counting Rbpms2-positive RGCs in young adult and aged killifish retinas using RGCode2 (De Schutter et al., 2025). Given that killifish undergo rapid retinal growth during the first 12 weeks—driven by both cell addition and tissue stretching (Bergmans et al., 2023b)—RGC densities after injury were normalized to their relative age-match uninjured controls (Figures 2A,B), as previously described (De Schutter et al., 2025). This natural expansion results in a higher total RGC count in aged fish (~135,000 vs. ~75,000 RGCs in young adults) despite a lower cell density (~14,000 vs. ~17,500 cells/mm^2^ in young adults), in line with previous observations (De Schutter et al., 2025). Following cONT, both young adult and aged fish exhibited substantial RGC loss, retaining only ~25% of RGCs by 21 dpi which persisted until 65 dpi (Figures 2A–C)—a loss rate comparable to that observed in murine optic nerve injury models (Tran et al., 2019; Masin et al., 2021). Intriguingly, early RGC loss dynamics differed between age groups: at 10 dpi, aged killifish retained ~70% of their RGCs, whereas young adults only retained ~50% (Figures 2A–C). This difference in early RGC survival may reflect a higher baseline number of RGCs in aged retinas or may result from extrinsic factors modulating the severity of secondary injury.

Because immune responses affect neuronal survival (Bosak et al., 2018; Chen et al., 2024), we examined microglia/macrophage dynamics after cONT using immunostaining for Lcp1, a pan-leukocyte marker, and quantified their densities both as absolute values and as fold-changes relative to the uninjured condition. Consistent with prior reports (Vanhunsel et al., 2021; Bergmans et al., 2024), aged uninjured retinas exhibited higher baseline immune cell densities than young adults (Figures 2D,E), a sign of inflammaging. Following cONT, 6-week-old fish exhibited a rapid immune response, with microglia/macrophage density peaking fourfold over baseline at 4 dpi (Figure 2F) and displaying a reactivated, amoeboid/rounded morphology (Figure 2D). At this timepoint, the number of immune cells was higher in young adult fish compared to aged counterparts (Figures 2D,E). In young adults, immune cell numbers then declined, returning to baseline levels by 21 dpi. In contrast, aged fish displayed a delayed response, with densities doubling only by 21 dpi—reaching levels comparable to the 4 dpi peak observed in young adults (Figures 2D–F). These results reveal age-specific neuroinflammatory profiles: a rapid, transient response in young adults versus a delayed response in aged fish. Despite these temporal differences, the peak magnitude of immune activation in absolute terms was comparable across age groups (Figures 2D,E), suggesting that the timing, rather than the extent, of inflammation may underlie differences in early RGC survival.

Overall, both young adult and aged killifish experience substantial RGC loss following cONT, mirroring survival rates observed in the murine retina, with the immune system likely playing a key role in modulating survival dynamics.

### 3.3 Extensive gliofibrotic scarring after cONT

**Figure 3:**
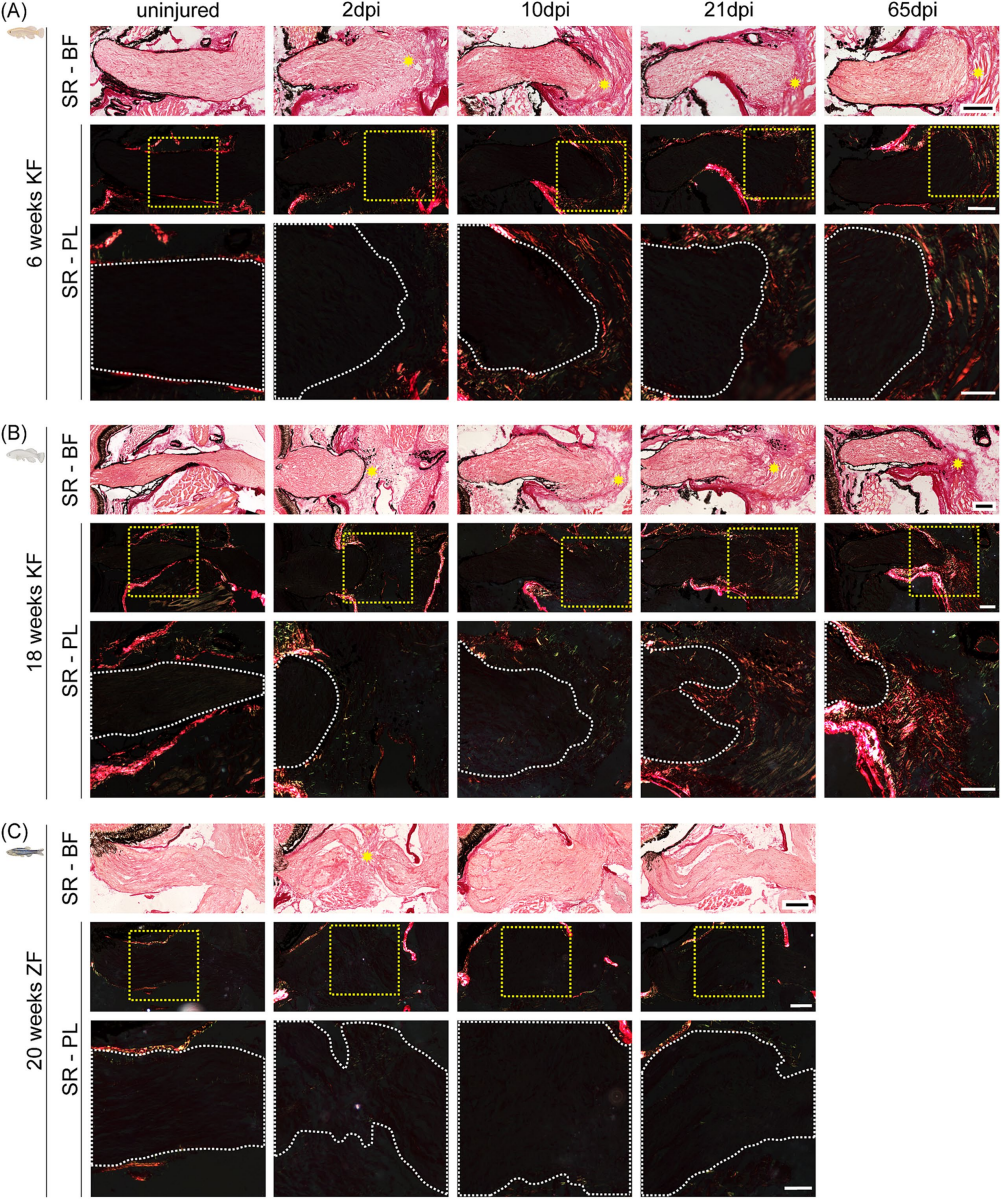
Species-specific and age-dependent scar formation and collagen deposition after cONT. **(A–C)** Representative Sirius red–stained longitudinal optic nerve sections after cONT in young adult killifish [6-week-old; **(A)**], aged killifish [18-week-old; **(B)**], and adult zebrafish [20-week-old, **(C)**]. Each panel shows the full nerve imaged under bright-field (BF), with the injury site marked by a yellow asterisk, and under polarized light (PL), where the yellow dotted outline indicates the region shown magnified below. Insets are magnified PL views with the optic nerve border demarcated by a white dotted line. With assets from Biorender.com. Scale bar optic nerve overview = 100 μm, scale bar zoom boxes = 50 μm. **(A)** In young adult killifish, a loose scar forms by 2 dpi, progressively consolidates into a dense structure by 10 dpi, and becomes compacted and enwrapped by muscle tissue by 21 dpi. By 65 dpi, the scar has increased in size and completely encapsulates the optic nerve stump. Collagen deposition, visible under PL, increases and matures over time, shifting from predominantly green (immature) at 10 dpi to red (mature) at 65 dpi (*N* = 6 per condition). **(B)** Aged killifish show a similar temporal pattern of scar formation and collagen maturation but develop a more extensive scar by 65 dpi compared to young adults (*N* = 3 per condition). **(C)** In contrast, adult zebrafish show no evidence of scar formation or collagen deposition after cONT. Instead, the optic nerve fully regenerates, restoring an uninjured-like morphology by 21 dpi (*N* = 6 per condition). BF, bright-field; cONT, complete optic nerve transection; dpi, days post injury; KF, killifish; PL, polarized light; SR, Sirius red; ZF, zebrafish.

Given the striking resemblance between the killifish and mammalian injury responses, we hypothesized that glial and fibrotic scarring might underlie the observed failure of axonal regeneration and tectal reinnervation, as it does in the mammalian CNS (Andries et al., 2021; Liu et al., 2024). To explore this, we performed Sirius red and H&E staining on longitudinal visual system cryosections from both young adult and aged killifish at multiple timepoints post-injury. In young adult killifish, bright-field. images of H&E (Supplementary Figure S2A) and Sirius red-stained (Figure 3A) optic nerves revealed a progressive scarring response: at 2 dpi, the proximal stump was surrounded by loosely organized cells, which by 10 dpi consolidated into a densely structured scar, encapsulating the optic nerve stump (Figure 3A; Supplementary Figure S2A). Over time, this scar became enwrapped by muscle tissue, forming a compact structure likely impeding axonal regrowth (Figure 3A; Supplementary Figure S2A). Aged killifish exhibited a comparable temporal pattern of scar formation and muscle encapsulation but developed a more extensive scar by 65 dpi (Figure 3B; Supplementary Figure S2B). To further characterize the extracellular matrix composition of the scar, we focused on two known extracellular components of the mammalian glial/fibrotic scar, namely collagen and fibronectin. Polarized light imaging of Sirius red-stained sections further showed that scarring coincided with collagen deposition and maturation, reflected by a color shift from green (immature collagen) to red (mature collagen) over time in both young adult and aged killifish (Figures 3A,B). This gradual accumulation of mature collagen ultimately resulted in a dense, collagen-rich barrier preventing tectal reinnervation (Figures 3A,B). Although the temporal timeline of scar formation was similar between both age groups, a visibly more mature collagen was observed in aged killifish at 65 dpi. *In situ* HCR for fibronectin (*fn1b*) revealed an increased number of *fn1b* expressing cells in the scar after cONT, in both young adult and aged killifish (Supplementary Figure S3). While in young adults these cells are already evident at 4 dpi, they become slightly more pronounced from 10 dpi onward (Supplementary Figure S3A). Aged killifish, on the other hand, already show a substantial *fn1b* expressing cell density from 4 dpi onward (Supplementary Figure S3B). The presence of *fn1b* expressing cells within the scar, which likely secrete fibronectin, suggest a potential role of fibronectin—next to collagen—in extracellular matrix remodeling following cONT, similar to the mammalian glial/fibrotic scar (Orr and Gensel, 2018).

As these findings contrasted the response observed after ONC in killifish, and to exclude possible procedural artifacts, we performed cONT in adult zebrafish, a species known for its robust regenerative capacity and successful tectal reinnervation post-ONT (Zou et al., 2013; Shen et al., 2025). Of note, anatomical differences of the optic nerves between the two teleost species are evident. While zebrafish display distinct axonal bundles (Figure 3C; Supplementary Figure S2C), killifish present with a more disorganized architecture (Figures 3A,B; Supplementary Figures S2A,B), akin to the murine optic nerve (Diekmann et al., 2015; Tzekov et al., 2016). After injury, neither Sirius red nor H&E staining revealed any evidence of scarring in zebrafish (Figure 3C; Supplementary Figure S2C). By 10 dpi, the proximal and distal stumps had reconnected, showing only mild swelling, and by 21 dpi, the optic nerve exhibited a morphology indistinguishable from uninjured tissue, with restored axonal bundling in the proximal region of the optic nerve (Figure 3C; Supplementary Figure S2C). To further confirm RGC axonal regrowth and tectal reinnervation, we used the *Tg(Tru.gap43:eGFP)* reporter line to trace regenerating axons. As expected, the *gap43* promoter was silenced under uninjured conditions in RGCs but strongly activated following cONT (Supplementary Figure S4). At 2 dpi, regenerating eGFP-positive axons were detected in the proximal optic nerve, with limited labeling in its distal segment and minor tectal reinnervation. By 10 dpi, eGFP labeling revealed a fully stained optic nerve, with nearly all regenerating axons extending across the lesion site to reinnervate the optic tectum (Supplementary Figure S4, yellow arrowheads). By 21 dpi, the complete RGC target layer of the optic tectum was reinnervated (Supplementary Figure S4, yellow arrowheads).

**Figure 4:**
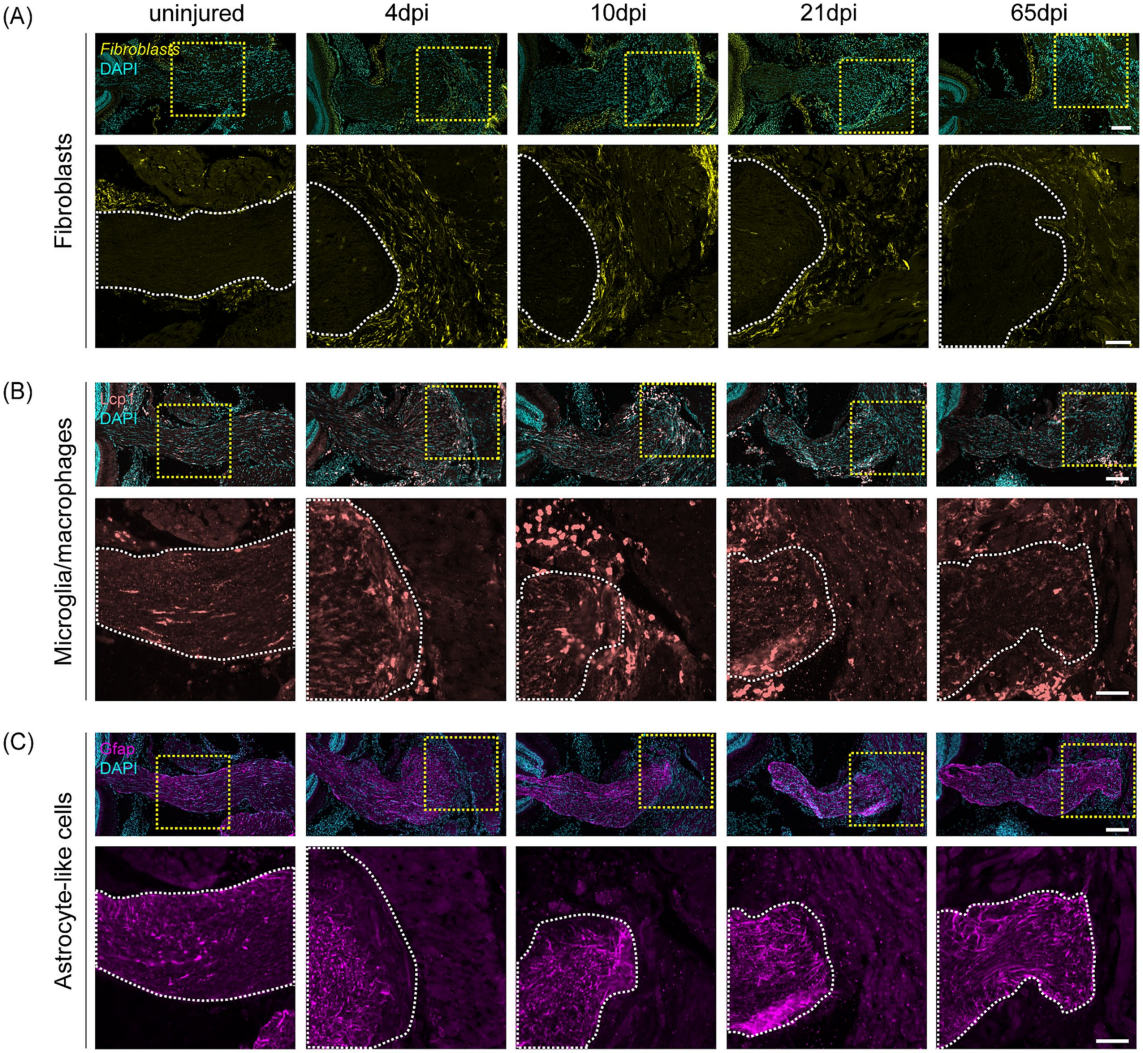
Fibrotic, immune, and gliotic response after cONT in young adult killifish. **(A–C)** Representative micrographs of complete optic nerve sections of young adult (6-week-old) killifish after cONT, showing different cell types. The yellow dotted outline marks the region shown magnified below, with the optic nerve border indicated by a white dotted line (*N* = 6 per condition). Scale bar optic nerve overview = 100 μm, scale bar zoom boxes = 50 μm. **(A)** Representative HCR images visualizing fibroblasts labeled with probe pairs for *lum* and *col1a1a* show that fibroblasts (yellow) are the predominant cell type within the scar, persisting throughout the examined post-injury period (4–65 dpi). **(B)** Microglia/macrophage (pink) recruitment, revealed by immunostaining for Lcp1, is evident, with a clear inflammatory response evoked after cONT within the optic nerve and invasion into the scar tissue at 10 dpi. Their presence within the optic nerve slightly declines by 21–65 dpi, while they remain highly enriched in the scar. **(C)** Astrocyte-like cells (magenta), shown via immunostaining for Gfap, become reactivated after cONT (increased Gfap signal compared to uninjured), a phenotype that persists from 4 up to 65 dpi. These reactivated cells form a distinct border within the optic nerve, lining the injury site. *col1a1a*, collagen type 1 alpha 1a; cONT, complete optic nerve transection; DAPI, 4′,6-diamidino-2-phenylindole; dpi, days post injury; Gfap, Glial Fibrillary Acidic Protein; HCR, *in situ* hybridization chain reaction; Lcp1, Lymphocyte Cytosolic Protein; *lum*, lumican.

Having established that the killifish optic nerve forms a dense, collagen-rich scar following cONT, we next sought to dissect the cellular composition of this scar in young adult killifish. To this end, we employed HCR and immunohistochemistry to detect fibroblasts, microglia/macrophages, and astrocyte-like cells. Fibroblast, identified by *lum* and *col1a1a* HCR probes, were rare in uninjured optic nerves, but became highly abundant within the lesioned tissue from 4 dpi onwards, coinciding with scar compaction and maturation (Figure 4A; Supplementary Figure S5A). By 10–21 dpi, fibroblasts densely populated the lesion core and persisted until 65 dpi, suggesting they are the dominant scar-resident cell type and primary source of collagen production. Leukocytes, including microglia and macrophages, were visualized via Lcp1 immunostaining (Figure 4B), and *apoeb* mRNA expression was used as a more specific microglial marker (Supplementary Figure S5B). Initially scattered throughout the uninjured nerve at relatively low densities, these cells accumulated at the proximal stump by 4–10 dpi and progressively infiltrated the maturing scar between up to 21 dpi (Figure 4B; Supplementary Figure S5B), presenting with a reactivated, amoeboid/rounded morphology (Figure 4B). By 65 dpi, microglia/macrophage density slightly decreased in the proximal stump, though remaining more abundant compared to uninjured conditions. At this timepoint, small populations of Lcp1-positive cells continued to be present within the scar (Figure 4B). Astrocyte-like cells, identified by Gfap immunolabeling (Figure 4C) and *cx43* mRNA expression (Supplementary Figure S5C), became increasingly prominent at the scar edges from 4 dpi onwards and persisted until 65 dpi (Figure 4C; Supplementary Figure S5C), displaying a reactive phenotype characterized by elevated Gfap expression (Figure 4C). These cells remained confined to the optic nerve stump, forming a continuous border that surrounded—but did not invade—the scar core (Figure 4C; Supplementary Figure S5C), consistent with astrocyte barrier formation described in the injured murine optic nerve (Andries et al., 2021).

Collectively, our findings demonstrate that the dense, collagen-rich scar formed after optic nerve transection in killifish is not a procedural artifact but a species-specific injury response. The predominance of collagen and fibronectin expressing fibroblasts within the scar, along with contributions from glial and immune cells, suggests that a gliofibrotic scar, characterized by extensive fibrosis and astrocyte-like border formation, constitutes a major barrier to axonal regeneration and target reinnervation in this animal model. Remarkably, this gliofibrotic scar recapitulates key cellular and extracellular matrix features of post-injury scarring in the mammalian CNS.

### 3.4 Successful target reinnervation after partial optic nerve transection (pONT) in young adult killifish

**Figure 5:**
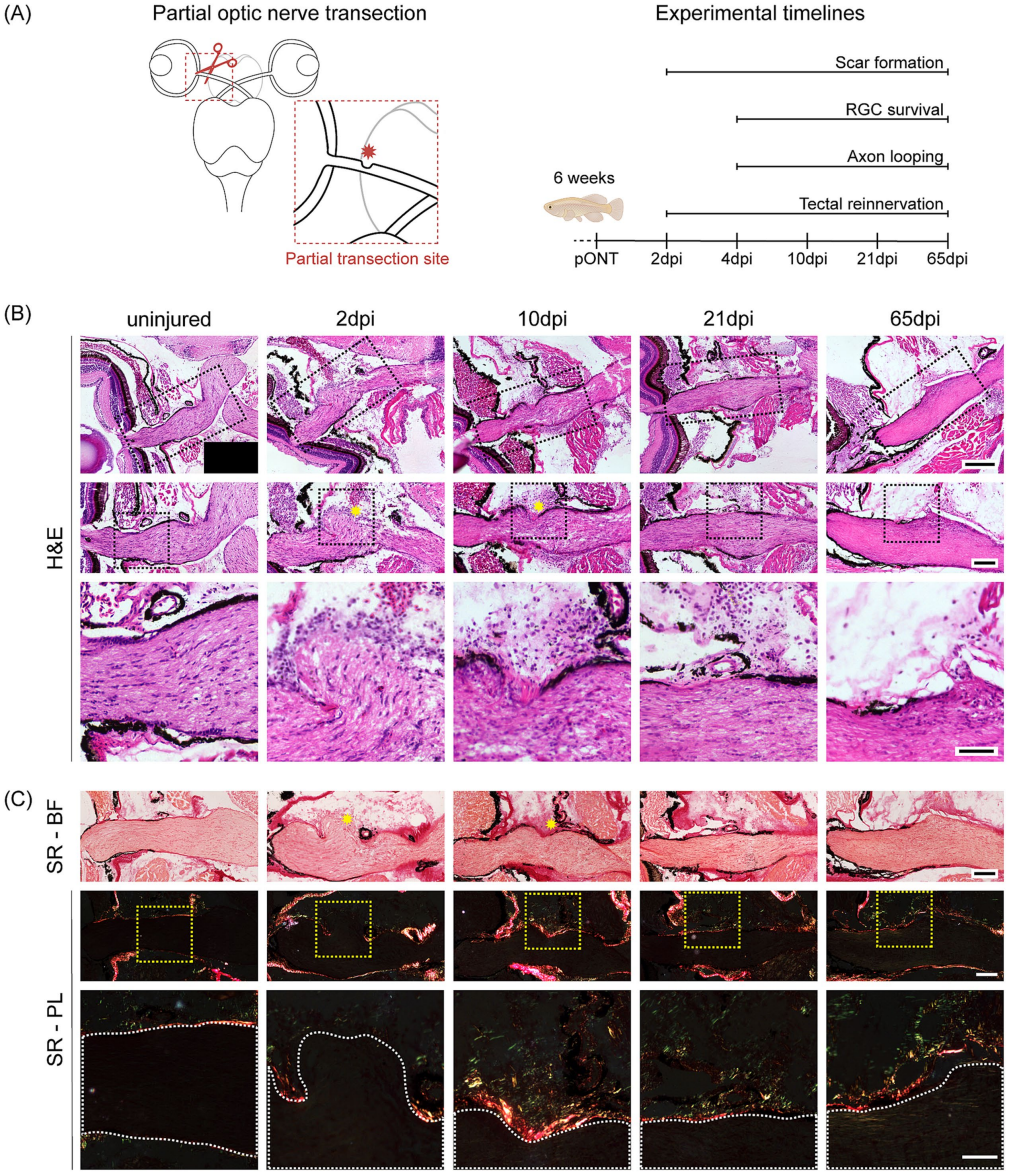
Partial optic nerve transection (pONT) prevents severe scar formation in young adult killifish. **(A)** Schematic overview of the pONT injury paradigm (dorsal view of visual system) and experimental timeline used to assess axonal regeneration in young adult (6-week-old) killifish. With assets from Biorender.com. **(B)** Representative H&E-stained visual system sections and corresponding zoom boxes showing the complete optic nerve and injury site after pONT (black dotted lines mark the zoomed views; *N* = 6 per condition). At 2 and 10 dpi, a partial lesion is visible (yellow asterisk), which gradually repairs, becoming negligible by 21 dpi. By 65 dpi, the optic nerve displays an uninjured-like morphology. Scale bar visual system = 200 μm, scale bar optic nerve overview = 100 μm, scale bar zoom box = 50 μm. **(C)** Representative Sirius red–stained optic nerve sections with injury-site magnifications (yellow dotted lines indicate the zoomed views; optic nerve outlined in white; *N* = 6 per condition) are shown following pONT, imaged under BF and PL. BF images show the transient injury at 2 and 10 dpi (yellow asterisk), which resolves by 65 dpi. PL reveals (im)mature collagen surrounding—but external to—the nerve from 2 dpi onward and most prominent at 10 dpi, with only a few fibers detected within the optic nerve at 65 dpi, aligned parallel to RGC axons. Scale bar optic nerve overview = 100 μm, scale bar zoom box = 50 μm. BF, bright-field; dpi, days post injury; H&E, hematoxylin and eosin; pONT, partial optic nerve transection; PL, polarized light; RGC, retinal ganglion cell; SR, Sirius red.

Our findings so far indicate that dense collagen-rich fibrotic scarring impedes successful target reinnervation in both young adult and aged killifish. To minimize scar formation and promote axonal regeneration, we next performed a pONT in young adult killifish and collected samples at multiple timepoints post-injury to evaluate the regeneration process (Figure 5A). This injury paradigm was designed to mimic surgical conditions in human optic nerve or eye transplantation, where donor and recipient nerve stumps are closely apposed—often stabilized by vascular remnants or surgical adhesives (Sabongi et al., 2015; Sallam et al., 2022)—and where limiting scar formation may facilitate axonal regrowth.

We first assessed whether pONT attenuates scar formation, as hypothesized, using H&E and Sirius red staining (Figures 5B,C). At 2 dpi, a well-defined lesion site was evident (Figures 5B,C, yellow asterisk), with partial preservation of optic nerve continuity. Over time, nerve architecture progressively recovered, with minimal fibrosis despite a local cellular response near the injury site. By 65 dpi, H&E and Sirius red staining revealed an optic nerve morphology largely comparable to uninjured tissue, with only a mild increase in cellular density at the original lesion (Figures 5B,C). Polarized light imaging of Sirius red-stained sections showed only sparse collagen fibers restricted to the original lesion site within the optic nerve at 65 dpi, aligned parallel to the RGC axons (Figure 5C). In contrast, the perineural region displayed a limited (im)mature collagen deposition starting at 2 dpi, peaking at 10 dpi (Figure 5C), similar to cONT (Figure 3A). However, unlike after cONT (Figure 3A), the perineural collagen response following pONT was less pronounced and gradually declined again over time (Figure 5C).

To further characterize the cellular response after pONT, we visualized the three major cell types previously implicated in scar formation upon cONT using HCR. Consistent with the minimal scarring observed histologically, the cellular response after pONT was overall milder and transient, but the response time to injury was similar to cONT, starting by 2 dpi for both injury paradigms (Figures 3–5). Fibroblasts (*lum^+^, col1a1a^+^*) accumulated transiently in the perineural region near the lesion, peaking at 4 dpi and returning to baseline by 65 dpi (Supplementary Figure S6A). Unlike the persistent fibroblast presence dominating the scar after cONT, their activation was limited and short-lived. Microglia/macrophages (*apoeb*^+^) showed a similar transient pattern within the optic nerve, with accumulation at 4 dpi and a gradual decline to baseline by 65 dpi (Supplementary Figure S6B). Only a sparse number of immune cells were detected near the lesion external to the nerve between 4–21 dpi (Supplementary Figure S6B, yellow arrowheads). Astrocyte-like cell (*cx43^+^*) involvement was modest and short-lived (10–21 dpi), lacking the dense border formation typical for cONT, and fully normalized again by 65 dpi (Supplementary Figure S6C). Together, these results indicate that although the initial scarring response is comparable between cONT and pONT models, its extent and persistence differ, with a weaker and more transient response following pONT.

**Figure 6:**
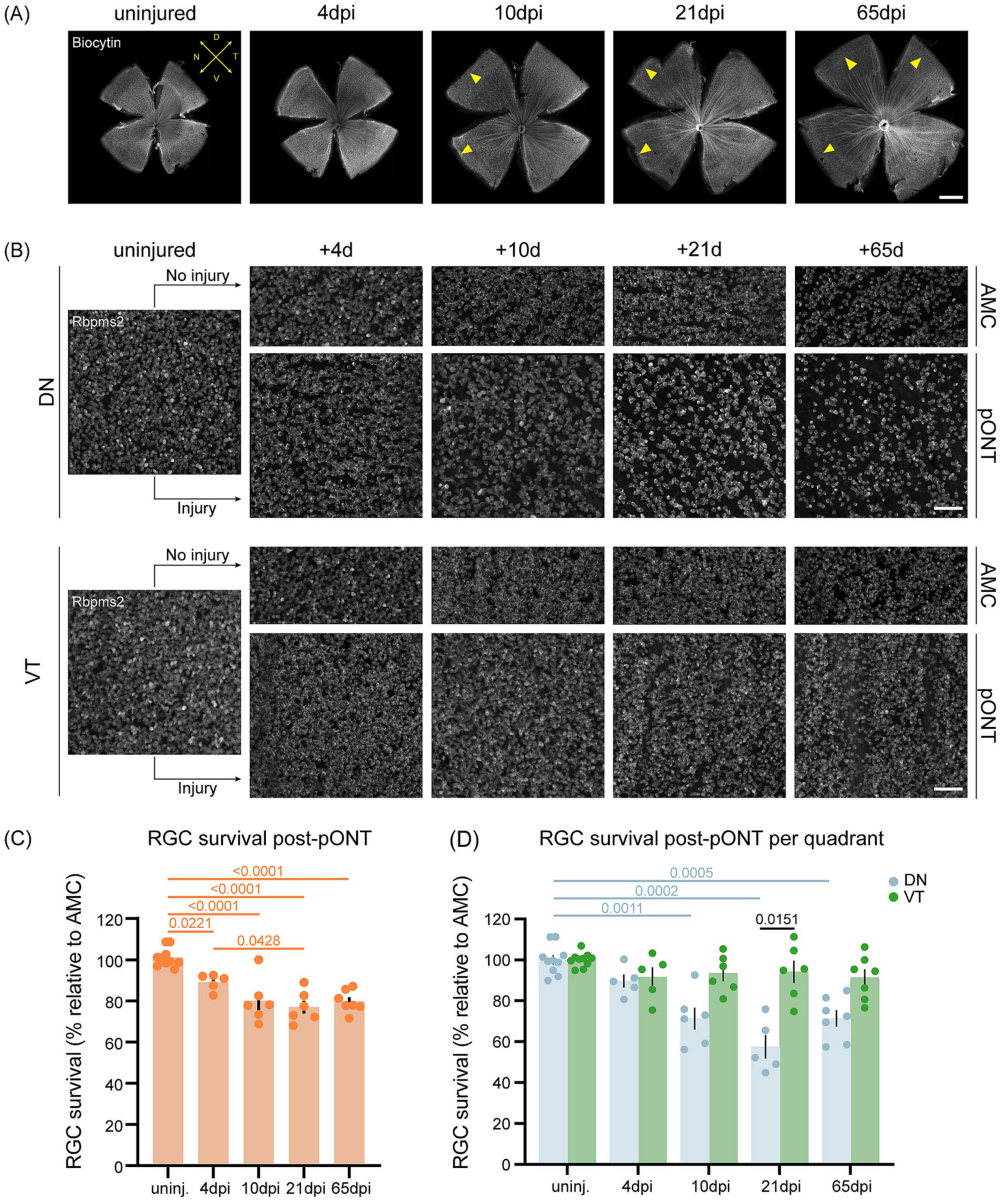
Minimal aberrant axonal growth and improved RGC survival after pONT in young adult killifish. **(A)** Representative biocytin-stained micrographs of retinal WMs of retrogradely traced young adult (6-week-old) killifish (*N* ≥ 5 per condition) showing very minimal aberrant RGC axon looping at the retinal margin from 10 dpi onwards (yellow arrowheads). Scale bar = 500 μm. **(B)** Representative micrographs of retinal WMs immunostained for Rbpms2 showing the dorsonasal (B) and ventrotemporal quadrants **(C)** from uninjured and injured (pONT) young adult killifish (*N* = ≥ 5 per condition). The uninjured retina displays regional RGC density differences, with higher RGC density in the ventrotemporal quadrant compared to dorsonasal. After pONT, a pronounced loss of RGCs is observed in the dorsonasal quadrant, corresponding to the injured optic nerve region, whereas the ventrotemporal quadrant, corresponding to the uninjured region, shows no evident cell loss. Scale bar = 50 μm. **(C)** Quantification of total RGC survival in Rbpms2-stained WMs shows a progressive decline starting from 4 dpi, retaining ~80% of RGCs at 65 dpi. Statistical analysis: two-way Kruskal–Wallis ANOVA followed by Mann–Whitney U tests with Bonferroni correction (*N* ≥ 5 per condition, presented as mean ± SEM). Significant differences (*p* < 0.05) are indicated within the graph. **(D)** Quadrant-based quantification reveals significant RGC loss in the DN quadrant by 10 dpi, with ~70% of dorsonasal RGCs remaining at 65 dpi. In contrast, the ventrotemporal quadrant shows minimal loss (~5% by 65 dpi). Statistical tests as in **(D)**. Blue *p*-values denote differences between timepoints in the dorsonasal quadrant, and black *p*-values between quadrants at specific timepoints. AMC, age-matched controls; d, days; D, dorsal; DN, dorsonasal; dpi, days post injury; DT, dorsotemporal; N, nasal; pONT, partial optic nerve transection; RGC, retinal ganglion cell; V, ventral; VN, ventronasal; VT, ventrotemporal; SEM, standard error of the mean; T, temporal, WM, whole-mount.

To determine whether intra-retinal axonal misrouting occurred independently of significant scar formation, regenerating RGC axons were retrogradely traced. At 10 dpi, only a few looping axons were found at the retinal margin (Figure 6A, yellow arrowheads). By 21 and 65 dpi, this phenotype became slightly more pronounced, likely reflecting the continuous looping of the small subset of misrouted RGC axons, but remained markedly less prominent compared to post-cONT conditions (Figures 1C, 6A). Furthermore, whole-retina quantification of RGC survival after pONT revealed high preservation rates with approximately ~80% of RGCs remaining at 65 dpi (Figures 6B,C), similar to survival levels after ONC (De Schutter et al., 2025). Regional analysis confirmed difference in RGC densities across quadrants of the uninjured retina with the ventrotemporal (VT) having the highest RGC density [~20,000 vs. 17,000 cells/mm^2^ in the dorsonasal (DN) quadrant; Figure 6B], as described before (De Schutter et al., 2025). Following pONT, RGC loss was largely confined to the directly injured DN quadrant, whereas the uninjured VT region remained mostly unaffected (Figure 6B). Quantitatively, the DN quadrant retained ~70% of its RGCs, while the VT quadrant showed only ~5% loss (Figure 6D). Thus, pONT—affecting roughly half of all RGCs— resulted in a disproportionately high degree of neuronal preservation compared with the widespread degeneration observed after cONT, evidenced by the fact that the injured DN retinal quadrant showed a relatively lower RGC loss compared to cONT.

**Figure 7:**
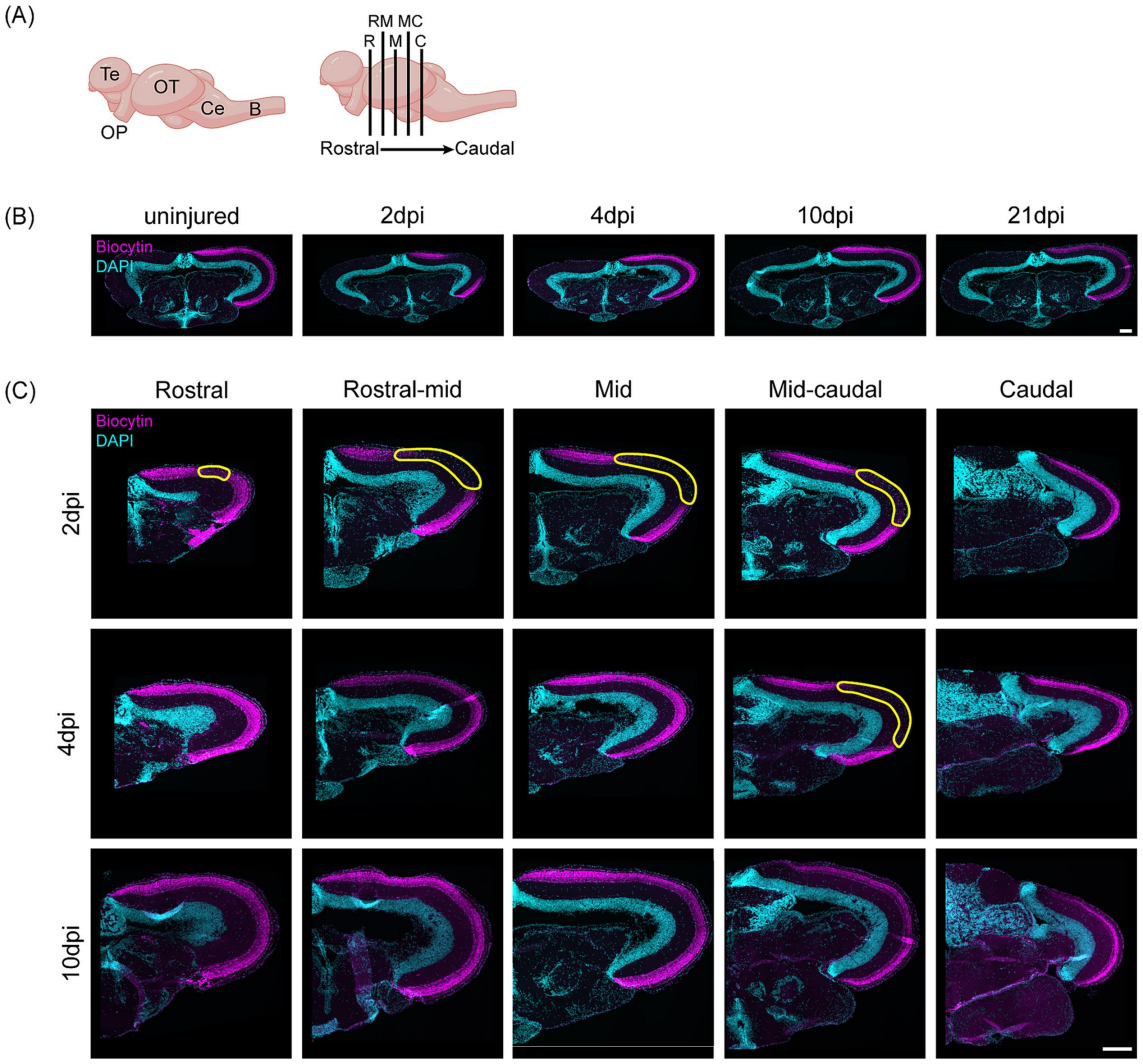
Successful target reinnervation after pONT in young adult killifish. **(A)** Schematic side view of the killifish brain illustrating the sectioning planes used to assess tectal reinnervation. **(B)** Representative biocytin-stained mid-coronal brain sections from anterogradely traced young adult (6-week-old) killifish after pONT (*N* = 6 per condition). In the uninjured condition, biocytin-positive RGC axons (magenta) innervate their target layer in the OT, whereas a distinct denervated zone is evident in the mid-tectal region at 2 dpi, which becomes fully reinnervated by 4 dpi. Scale bar = 200 μm. **(C)** Representative series of rostral-to-caudal images of the right tectal hemisphere from anterogradely traced young adult (6-week-old) killifish after pONT. At 2 dpi, the rostral to mid-caudal regions of the tectum show extensive denervation, which is largely restored by 4 dpi, although the mid-caudal region remains partially denervated (yellow outlines). By 10 dpi, the entire optic tectum is successfully reinnervated. Scale bar = 200 μm. B, Brainstem; C, caudal; Ce, cerebellum; DAPI, 4′,6-diamidino-2-phenylindole; M, mid; MC, mid-caudal; OP, optic projection; OT, optic tectum; pONT, partial optic nerve transection; R, rostral; RM, rostral-mid; Te, telencephalon.

Given the lack of gliofibrotic scarring, minimal intra-retinal axon looping, and high RGC survival observed after pONT, we next examined whether young adult killifish could successfully reinnervate their optic tecti. Biocytin labeling, post-anterograde tracing, on mid-coronal brain sections (Figure 7A) revealed a clear biocytin-negative zone in the optic tectum—devoid of RGC terminals—at 2dpi (Figure 7B). By 4 dpi, this tectal region was already largely reinnervated (Figure 7B). Evaluation of the reinnervation pattern across the rostro-caudal axis at 2, 4 and 10 dpi revealed that the tectum showed widespread denervation at 2dpi, except for the most caudal section (Figures 7A–C). By 4 dpi, reinnervation was nearly complete, with only a small mid-caudal area remaining devoid of RGC terminals (Figure 7C, yellow lining). At 10 dpi, the optic tectum was fully reinnervated along the entire rostro-caudal axis (Figure 7C), demonstrating the rapid and robust regenerative capacity of young adult killifish post-pONT, i.e., in the absence of a gliofibrotic scar.

Together, these findings demonstrate that young adult killifish are capable of complete target reinnervation after pONT, accompanied by limited RGC loss and minimal aberrant intra-retinal growth. The absence of a prominent collagen-rich fibrotic scar and astrocyte-like cell border delineating the injury site likely permits this regenerative success, emphasizing the inhibitory effect of fibrotic scarring and astroglial bordering, thereby highlighting the importance of the extra-neuronal milieu in axon regeneration and potential whole-eye transplantation strategies.

## 4 Discussion and conclusion

The limited regenerative capacity of the human CNS makes retinal pathologies a persistent clinical challenge, with few effective therapies available to restore/preserve function. Whole-eye transplantation represents an exciting avenue toward vision restoration, although reinnervation of visual targets remains a major obstacle. The killifish offers a unique opportunity to study the mechanisms underlying regenerative success and failure due to its hybrid regeneration profile: young adults exhibit robust CNS regeneration, whereas aging leads to a gradual decline toward a mammalian-like, regeneration-impaired phenotype (Van Houcke et al., 2021; Vanhunsel et al., 2022a, 2022b). In this study, RGCs from both young adult and aged killifish failed to reinnervate their brain targets following cONT, in stark contrast to regeneration-competent zebrafish (Zou et al., 2013; Ali et al., 2025). Instead, regenerating axons formed aberrant loops at the retinal margin, which were less prominent in aged killifish, most likely reflecting their reduced intrinsic growth capacity (Vanhunsel et al., 2022a). The regenerative failure was accompanied by significant RGC loss (~75%) in both age groups, albeit with distinct temporal dynamics and age-dependent differences in retinal immune responses. Moreover, a dense, collagen-rich gliofibrotic scar rapidly formed at the lesion site, becoming more extensive in aged individuals at later post-injury stages. To mitigate scarring and enhance regeneration, we performed pONT in young adult killifish, which resulted in negligible scar formation, reduced axonal misrouting, improved RGC survival, and successful reinnervation of the optic tectum.

Research using lower vertebrates, particularly teleost fish, has significantly advanced our understanding of CNS repair. Species such as goldfish and zebrafish exhibit robust regenerative capacities, enabling them to recover from severe CNS injuries, including ONC, ONT, spinal cord injury, and traumatic brain injury (Becker et al., 1997; Becker and Becker, 2007, 2014; Zupanc, 2008; Diekmann et al., 2015; Rasmussen and Sagasti, 2017; Beckers et al., 2019, 2021; Cigliola et al., 2023; Shen et al., 2025). Following ONC or traumatic brain injury, young adult killifish are also able to functionally recover (Vanhunsel et al., 2022a; Mariën et al., 2025), while aged individuals exhibit severely reduced CNS repair and fail to regain function (Van Houcke et al., 2021; Vanhunsel et al., 2022a). While ONC has been well characterized in killifish (Vanhunsel et al., 2022a; De Schutter et al., 2025), it may be less suitable as a model for whole-eye transplantation. In this context, ONT represents a more relevant injury paradigm. Both ONC and ONT have been extensively studied in zebrafish and goldfish, showing successful regeneration in each case. However, ONT typically results in lower RGC survival and slower, but functional, recovery compared to ONC (Stuermer et al., 1992; McDowell et al., 2004; Zou et al., 2013; Diekmann et al., 2015).

In contrast to ONC (Vanhunsel et al., 2022a), adult killifish fail to reinnervate their brain targets following cONT, irrespective of age. This outcome is unexpected, given that RGCs in killifish are known to possess strong intrinsic regenerative capacity (Vanhunsel et al., 2022a). Following cONT, surviving killifish RGCs retain this growth potential; however, instead of projecting toward the brain, their axons are misrouted back into the retina, forming aberrant loops at the retinal margin within the inner plexiform layer, adjacent to the ganglion cell layer. This continued growth suggests that key intrinsic mechanisms—such as growth cone formation, polarization, and cytoskeletal reorganization—are functional in both young adult and aged killifish axons after cONT (Dent et al., 2011; Mar et al., 2014; Mahar and Cavalli, 2018). The observations point to alterations in the local retinal environment. A potential contributor to the disorganized yet permissive growth environment near the looping axons in the retina is the formation of a glial scaffold at the looping site. In zebrafish, growth-promoting glial scaffold formation has been described as crucial during injury-induced regeneration and axonal elongation (Goldshmit et al., 2012; Becker and Becker, 2014; Wehner et al., 2017; Cigliola et al., 2020, 2023; John et al., 2025).

Being trapped within the retina, killifish RGCs are unable to re-establish functional synapses with their brain targets. As previously reported, the absence of synaptic connectivity and consequent loss of brain-derived trophic support can critically impair RGC survival (Biehlmaier et al., 2001; Boia et al., 2020; De Schutter et al., 2025). Consistent with this, we observed substantial RGC loss post-cONT, with only ~25% of RGCs surviving at 21 dpi, a proportion that remained stable up to 65 dpi. This severe loss of RGCs exceeds the one observed post-ONC, where young adult and old killifish preserve ~65% and ~45% of their RGCs, respectively, at 21 dpi (De Schutter et al., 2025). Our findings support the existence of intrinsically resilient RGC subtypes in killifish that do not require synapse reformation nor brain-derived trophic support to survive, similar to subtype-specific survival responses described in the murine retina (Tran et al., 2019; De Schutter et al., 2025). Notably, older killifish (18-week-old) exhibited higher RGC survival at 10 days post-cONT compared to younger adults (6-week-old). Previous work using the ONC model has defined two distinct phases of RGC loss in killifish: an early phase likely reflecting injury-induced cell death (primary injury response), and a secondary phase linked to extrinsic factors such as immune-mediated mechanisms (secondary injury response) (De Schutter et al., 2025). Our data suggest that age-related differences in injury-induced immune responses may delay the onset of secondary RGC loss in aged fish. Following cONT, aged killifish exhibit a delayed peak in retinal immune cell infiltration (21 dpi versus 4 dpi in young adults), likely postponing secondary injury-mediated RGC loss. These findings highlight the importance of distinguishing between primary and secondary injury mechanisms in the context of CNS repair. Notably, the immune response after cONT in young adult killifish is markedly more severe than after ONC. Whereas ONC results in an approximately twofold increase in the Lcp1-immunopositive area in retinal sections (Vanhunsel et al., 2022a), cONT induces a far more extensive microglial response in the retina, with Lcp1-positive cells roughly quadrupling. Together with the pronounced RGC loss observed after cONT, these findings are consistent with a robust and sustained inflammatory response in the retina following cONT. Overall, killifish RGCs thus exhibit a hybrid regenerative profile, maintaining zebrafish-like regrowth potential while demonstrating mammalian-like neuronal vulnerability.

One of the major non-neuronal limitations to successful axonal regeneration in the mammalian optic nerve is glial and fibrotic scar formation, in which astrocytes border the fibrotic scar core (Andries et al., 2021; Liu et al., 2021; Jin et al., 2022). In line with this, and unlike the regenerative response observed in zebrafish (Cigliola et al., 2023; John et al., 2025; Shen et al., 2025), adult killifish develop a persistent, dense, collagen-rich fibrotic scar following cONT, which is bordered by astrocyte-like cells. Similar to the murine injury response (Andries et al., 2021; Liu et al., 2021; Jin et al., 2022), this gliofibrotic lesion is likely to constitute both a chemical and mechanical barrier that prevents axons from reinnervating their brain targets. By contrast, ONC in young adult killifish induces only a transient extracellular matrix response, with limited collagen deposition and absence of a persistent fibrotic scar (Vanhunsel et al., 2022a). Zebrafish do not form a persistent lesion barrier at all after optic nerve injury, whether by transection or crush (Zou et al., 2013; Becker and Becker, 2014; Beckers et al., 2021; Shen et al., 2025), although the formation of a growth-permissive glial bridge has been described following spinal cord injury (Goldshmit et al., 2012; Becker and Becker, 2014; Wehner et al., 2017). A single-cell RNA sequencing study of the zebrafish spinal cord after transection revealed that this glial bridge is composed of ependymo-radial glia, fibroblasts, and immune cells (John et al., 2025). Importantly, this structure is pro-regenerative: glial cells form a permissive bridge instead of a restrictive border, and fibroblasts contribute extracellular matrix components that support, rather than inhibit, axonal growth (Goldshmit et al., 2012; Wehner et al., 2017; Cigliola et al., 2020, 2023; John et al., 2025). Several studies highlight the importance of the immune system during successful spinal cord regeneration (Tsarouchas et al., 2018; Cavone et al., 2021), while a more recent study specifically emphasizes the immune-fibroblast crosstalk as a critical player in orchestrating this pro-regenerative environment (John et al., 2025). A transient, non-fibrotic fibroblast state, induced after spinal cord injury, is believed to play a key role in initiating the inflammatory response and coordinating neutrophil resolution, both crucial for functional regeneration (John et al., 2025). In killifish, we observe pronounced involvement of fibroblasts, microglia/macrophages and astrocyte-like cells during scar formation. Unlike zebrafish, however, killifish fibroblasts appear to adopt a more fibrotic inhibitory phenotype, microglia/macrophages are chronically activated, and reactivated astrocyte-like cells from a dense border at the nerve-scar intersection, mirroring the environmental mammalian CNS response to injury (Andries et al., 2021; Liu et al., 2021; Jin et al., 2022).

In an effort to reduce scar formation, we performed pONT. This strategy significantly attenuated fibrotic scarring and the restrictive bordering by astrocyte-like cells, mitigated RGC loss, and facilitated successful reinnervation of the brain. A possible explanation for the differential outcomes between complete and partial transection lies in the nature of the immune response. cONT induces a robust and sustained inflammatory reaction within the optic nerve, whereas the immune response following pONT is more subdued and transient, mirroring the chronic inflammation seen in mammals and the more regulated response observed in zebrafish, respectively (Goldshmit et al., 2012; John et al., 2025). Previous research has described clear correlations/causations between the immune system and the formation of either a permissive transient scar in zebrafish or an inhibitory astrocyte-bordered fibrotic scar in mammals (Goldshmit et al., 2012; Wehner et al., 2017; Andries et al., 2021; Liu et al., 2021, 2024; Jin et al., 2022; John et al., 2025), patterns that are recapitulated after cONT and pONT in killifish, respectively. These findings highlight the critical importance of controlling scar formation—including all its cellular components; fibroblasts, immune cells and astrocyte(−like cell)s—in the CNS as a prerequisite for effective repair. Future research should therefore aim to comprehensively characterize both the transcriptomic and proteome profiles of all these cells both after cONT and pONT, ideally within a cross-species comparative framework that includes zebrafish and mouse. Such analyses could identify (i) conserved wound-healing programs that correlate with regeneration failure and (ii) pro-regenerative programs associated with a permissive lesion environment. If conserved, these pathways could serve as therapeutic entry points in mammals, enabling pre-emptive modulation of the injury environment to prevent scar formation or reprogram it into a growth-permissive scaffold, as observed in zebrafish. This approach could bypass the need for enzymatic scar degradation, which only removes the physical barrier but fails to install a growth-permissive, regenerative niche. Of note, in mammals, axonal regeneration is further compounded by a poor intrinsic growth potential of CNS neurons, making both cell-intrinsic and -extrinsic factors key obstacles to functional recovery. Indeed, enhancing axonal growth competence remains a pivotal component of pro-regenerative strategies, which can be achieved through targeted modulation of signaling pathways such as mTOR and JAK/STAT or developmentally regulated transcription factors (He and Jin, 2016; Mahar and Cavalli, 2018).

In summary, our findings demonstrate that, following cONT, killifish mount a mammalian-like regenerative response marked by the formation of a dense, collagen-rich fibrotic scar at the lesion site, bordered by astrocyte-like cells. This bordered scar impedes reinnervation of brain targets, causes axonal misrouting, and leads to pronounced retinal neuronal loss. Attenuating scar formation markedly improved regeneration, ultimately restoring target reinnervation. These results position the killifish as a valuable model for dissecting the cellular mechanisms—particularly fibroblast-immune-astrocyte-like cell crosstalk during gliofibrotic scarring—that underlie regenerative failure in the CNS, offering direct relevance for developing effective strategies toward whole-eye transplantation.

## Data Availability

The original contributions presented in the study are included in the article/Supplementary material, further inquiries can be directed to the corresponding authors.

## Footnotes

1
https://gitlab.com/NCDRlab/easy_hcr

## Glossary

*apoeb*: apolipoprotein Eb
aTub: acetylated tubulin
Blbp: Brain Lipid-Binding Protein
CNS: central nervous system
*col1a1a*: collagen type 1 alpha 1a
cONT: complete ONT
*cx43*: connexin 43
DAPI: 4′,6-diamidino-2-phenylindole
DN: dorsonasal
dpi: days post-injury
*fn1b*: fibronectin 1B
Gfap: Glial Fibrillary Acidic Protein
H&E: hematoxylin and eosin
HCR: *in situ* hybridization chain reaction
IDT: integrated DNA technologies
Lcp1: Lymphocyte Cytosolic Protein 1
*lum*: lumican
ONC: optic nerve crush
ONT: optic nerve transection
PBS: phosphate-buffered-saline
PBS-DEPC: PBS-diethyl pyrocarbonate
PBST: PBS-triton
PFA: paraformaldehyde
PID: pre-immune donkey serum
pONT: partial ONT
Rbpms2: RNA Binding Protein with Multiple Splicing 2
RGC: retinal ganglion cell
ROI: region of interest
THEA: Total Human Eye Allograft
VT: ventrotemporal
WM: whole-mount
