# Supplementary material for: Fibrotic scarring prevents optic nerve regeneration despite preserved axonal growth potential in adult killifish
Source: Front Neurosci. 2026 Feb 18;20:1745022. doi: 10.3389/fnins.2026.1745022 (PMC12956645; doi:10.3389/fnins.2026.1745022)
Supplement: Supplementary file 1 [file Data_Sheet_1.pdf]

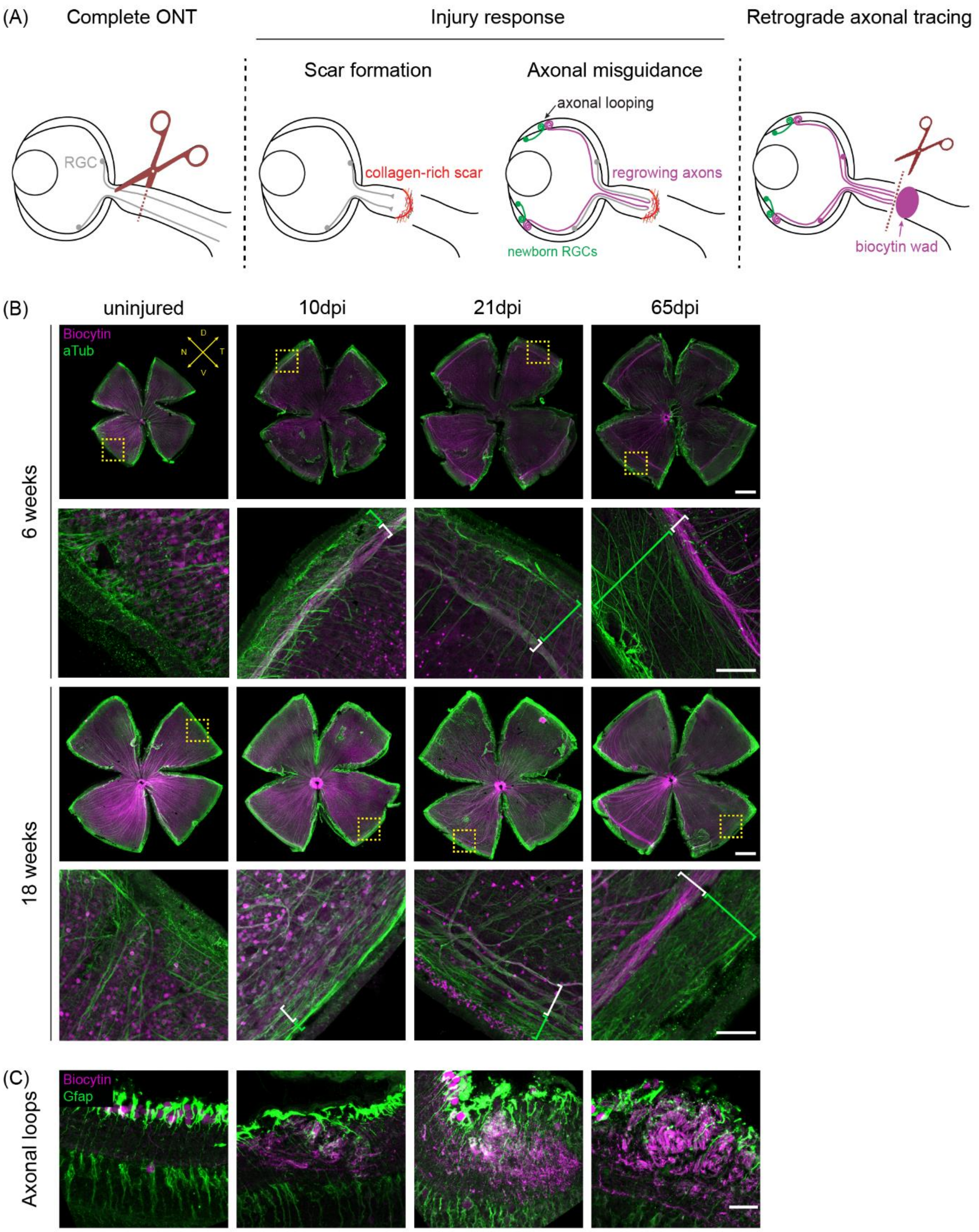

Supplementary figure S1: Axonal misguidance after cONT in the retina of young adult and aged killifish.

(A) Schematic overview of axonal misguidance upon cONT. Following cONT, a collagen-rich scar rapidly forms at the lesions site, creating a barrier that prevents regenerating RGC axons from extending towards their target brain area, the optic tectum. Upon encountering this barrier, axons make a U-turn, redirecting toward the eye, and continue to grow aberrantly within the retina, forming loop-like structures at the retinal margin. Axons sprouting from newly generated RGCs

also follow this aberrant growth trajectory and run directly adjacent to these retinal loops. Retrograde biocytin tracing, achieved by placing a biocytin-soaked wad at the lesion site, labels regenerating RGC axons and their somas. Regenerating axons and axons derived from newborn RGCs can be distinguished by their marker expression: regenerating RGC axons are double-positive for biocytin and acetylated tubulin (aTub), whereas axons from newborn RGCs are only immunopositive for aTub.

(B) Representative micrographs of retinal WMs stained for biocytin (magenta) and acetylated tubulin (aTub, green) from retrogradely traced young adult (6-week-old) and aged (18-week-old) killifish (N = ≥ 5 per condition). Zoomed images, indicated by yellow dotted outlines on complete retinal WMs, show clear biocytin- and aTub-positive axonal loops from regenerating RGCs (highlighted by white braces) near the retinal border after cONT. Adjacent, and more peripheral, to these regenerating axons are aTub-positive, but biocytin-negative axon bundles (indicated by green braces), likely sprouted from newborn RGCs. The extent of newborn RGC axons looping in the retina increases over time as more cells are added during its continuous growth. Aged fish show a less pronounced looping phenotype in both the regenerating (aTub- and biocytin-positive) and newborn (aTub-positive and biocytin-negative) RGC axons, consistent with reduced axonal regrowth and diminished neuroregenerative capacity. Scale bar retinal WM = 500 μm, scale bar zoom boxes = 50 μm.

(C) Representative high-magnification micrographs of looping axons on retinal sections from young adult killifish at different timepoints post-cONT, co-stained for biocytin (magenta) and Gfap (green, N = 6 per condition). Looping biocytin-positive axons, first evident at 10 dpi, are closely associated with a Gfap immuno-positive scaffold throughout the evaluated post-injury period, with maximal overlap observed at 10 and 21 dpi. By 65 dpi, Gfap-positive fibers extend through the axonal loops. Scale bar = 25μm.

Abbreviations: aTub, acetylated tubulin; cONT, complete optic nerve transection; DAPI, 4',6-diamidino-2-phenylindole; D, dorsal; dpi, days post injury; Gfap, Glial Fibrillary Acidic Protein; N, nasal; RGC, retinal ganglion cell; T, temporal; V, ventral; WM, whole-mount.

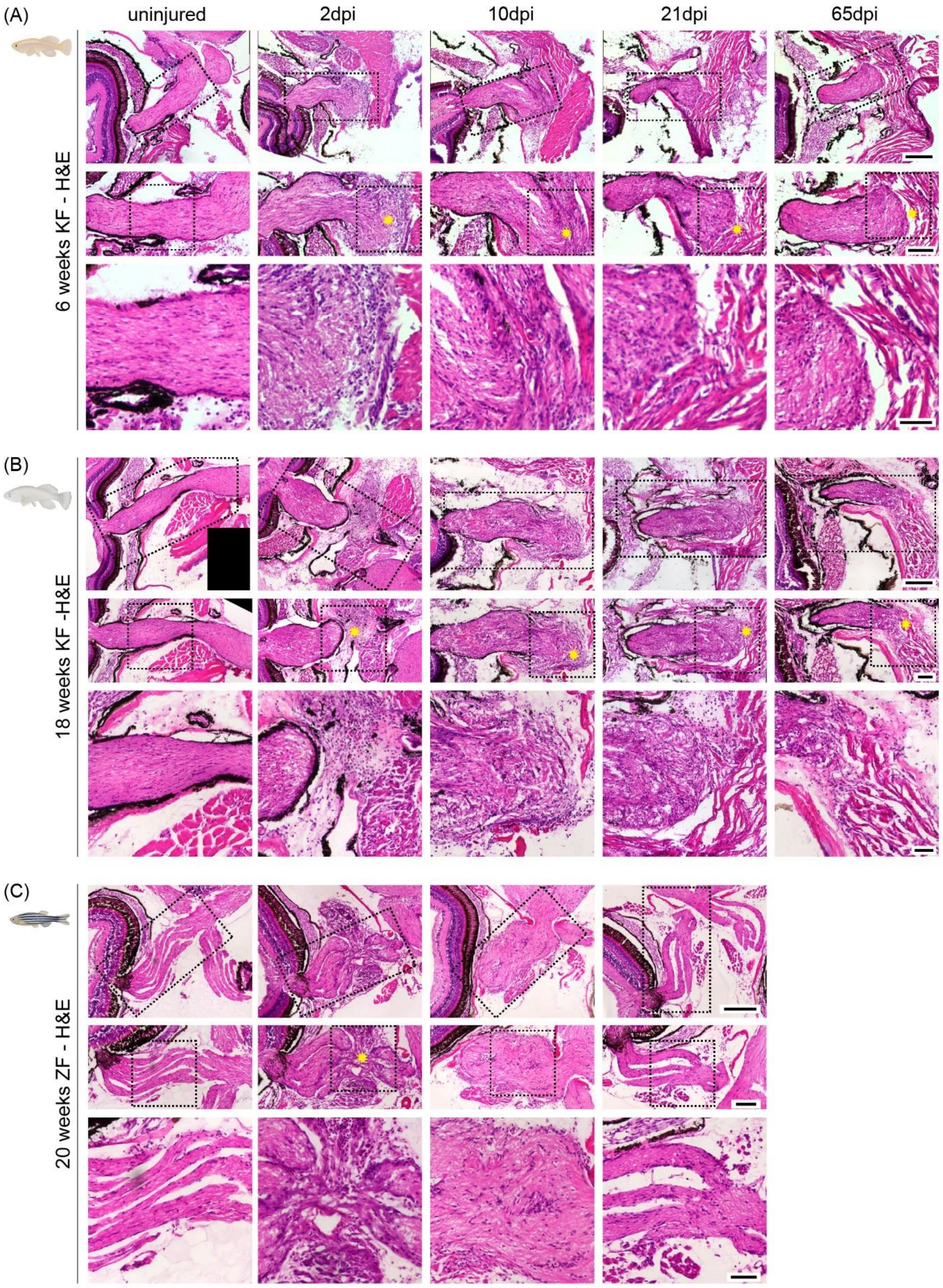

Supplementary figure S2: Scar formation after cONT.

(**A-C**) Representative H&E-stained visual system sections and corresponding zoom boxes showing the complete optic nerve and injury site (yellow asterisk) from young adult (6-week-old; A) and aged (18-week-old; B) killifish, and adult zebrafish (20-week-old; C) after cONT (black

dotted lines mark the zoomed views; N ≤ 6 per condition). With assets from Biorender.com. Scale bar visual system = 200 μm, scale bar optic nerve = 100 μm, scale bar zoom box = 50 μm.

(**A,B**) Both young adult and aged killifish exhibit clear scar formation following cONT. By 2 dpi, loosely dispersed cells surround the optic nerve stump, and progressively compact into a defined scar by 10 dpi. Over time, the scar increases in size and completely encapsulates the optic nerve stump. Of note, from 21 dpi onward, muscle tissue wraps the dense scar. Age killifish (B) develop a more extensive scar over time compared to young adults.

(**C**) In contrast, zebrafish do not form a scar upon cONT and display uninjured-like morphology by 21dpi. Of note, uninjured zebrafish optic nerves show clear axonal bundles, that start to reappear 21 days post-cONT.

Abbreviations: cONT, complete optic nerve transection; dpi, days post injury; H&E, hematoxylin and eosin; KF, killifish; ZF, zebrafish.

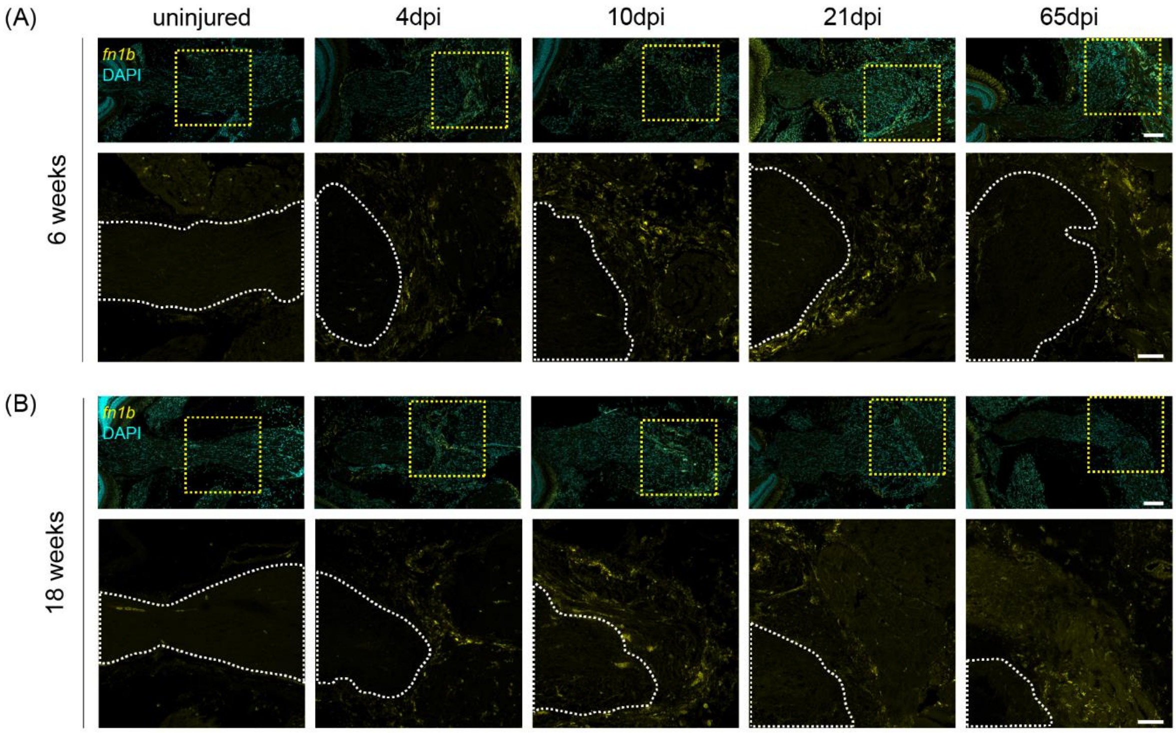

Supplementary figure S3: Presence of fibronectin expressing cells within the scar following cONT.

(**A-B**) Representative HCR images of complete optic nerve sections of young adult (6-week-old) and aged (18-week-old) killifish visualizing fibronectin (*fn1b*) expressing cells after cONT. The yellow dotted outline marks the region shown magnified below, with the optic nerve border indicated by a white dotted line (N = 6 per condition). Scale bar optic nerve overview = 100 µm, scale bar zoom boxes = 50 µm.

(**A**) In young adult killifish, *fn1b*-expressing cells are moderately present at 4dpi and become increasingly prominent from 10dpi onward.

(**B**) In aged killifish, a high density of *fn1b*-expressing cells is already observed at 4dpi and remains relatively consistent over time.

Abbreviations: cONT, complete optic nerve transection; DAPI, 4',6-diamidino-2-phenylindole; dpi, days post injury; *fn1b*, fibronectin 1B.

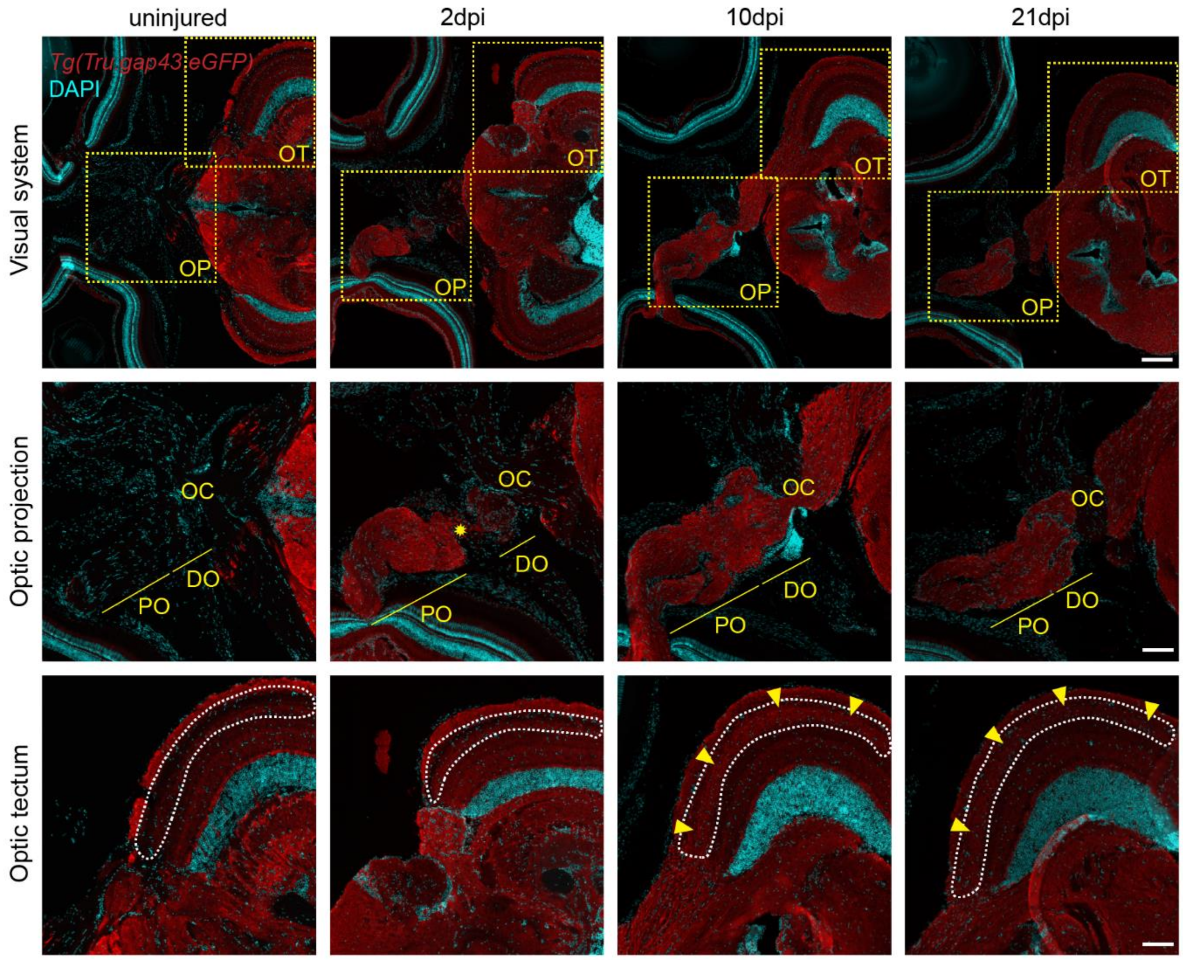

Supplementary figure S4: Successful axonal regeneration and tectal reinnervation after cONT in adult zebrafish.

Representative micrographs of eGFP-immunolabeled visual system sections from adult (20-week-old) *Tg(Tru.gap43:eGFP)* zebrafish showing eGFP expression (red) in regenerating RGC axons (N = 6 per condition). Viewed zooms (marked by yellow dotted squares) for both the optic projection and optic tectum are shown. Under uninjured conditions, the gap43 promoter remains inactive in retinal cells because no axons are regenerating, reflected by the absence of eGFP-positive axons in the optic nerve and in the predominant RGC tectal target layer (white dotted line). By 2 days post-cONT, the transection site is clearly visible (yellow asterisk), and eGFP signal is largely restricted to the proximal optic nerve, with only minimal labeling detected in the distal segment and optic tectum. From 10 dpi onward, eGFP positive RGC axons extend along the entire optic nerve, having passed the optic chiasm, and near fully reinnervate their target layer in the optic tectum (yellow arrowheads). Full tectal reinnervation is achieved by 21 dpi. Scale bar visual system = 200 µm, scale bar optic nerve and tectum = 100 µm.

Abbreviations: cONT, complete optic nerve transection; DO, distal optic nerve; dpi, days post injury; OC, optic chiasm; OP, optic projection; OT, optic tectum; PO, proximal optic nerve; RGC, retinal ganglion cell.

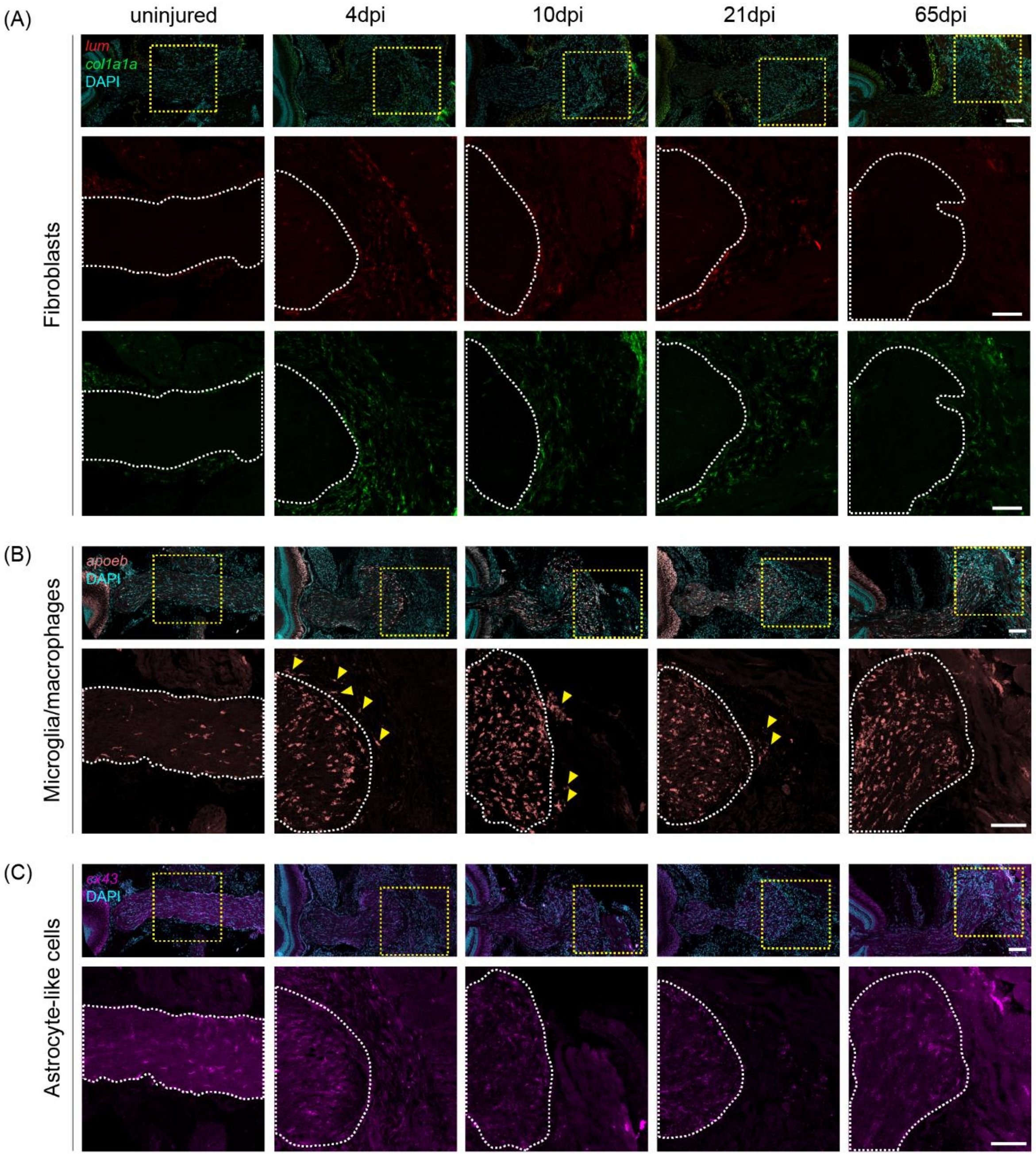

Supplementary figure S5: Fibroblast, immune and astrocyte-like cell responses after cONT in young adult killifish.

(**A-C**) Representative HCR images of complete optic nerve sections from young adult killifish (6-week-old) after cONT, visualizing fibroblasts, immune and astrocyte-like cells (N = 6 per condition). The yellow dotted squares indicate the region shown magnified below, with the optic nerve border demarcated by a white dotted line (N = 6 per condition). Scale bar optic nerve overview = 100 µm, scale bar zoom boxes = 50 µm.

(**A**) Fibroblasts are visualized using the probes for *lum* (red) and *col1a1a* (green). While their combined expression shows that they are the predominant cell type after cONT, persisting throughout the examined post-injury time window (see Figure 4A). Visualization of their individual expression pattern reveals that, in contrast to *col1a1a*-positive fibroblasts, *lum* expressing cells have disappeared from the scar region by 65 dpi.

(**B**) Microglia/macrophages (pink), visualized using probe pairs for *apoeb*, accumulate at the injury site within the optic nerve from 4 dpi, peak at 10 dpi, and mildly reduce in number between 21 and 65 dpi. Immune cells are also present within lesion site and the scar from 4 to 21 dpi (yellow arrowheads), but are largely resolved by 65 dpi.

(**C**) Astrocyte-like cells (magenta), labeled with probe pairs for *cx43*, are confined to the optic nerve, bordering the scar. Of note, mild non-specific background staining is present but remains clearly distinguishable from the astrocyte-like morphology of the *cx43*-positive cells.

Abbreviations: *apoeb*, apolipoprotein Eb; *col1a1a*, collagen type 1 alpha 1a; cONT, complete optic nerve transection; *cx43*, connexin 43; DAPI, 4',6-diamidino-2-phenylindole; dpi, days post injury; *lum*, lumican.

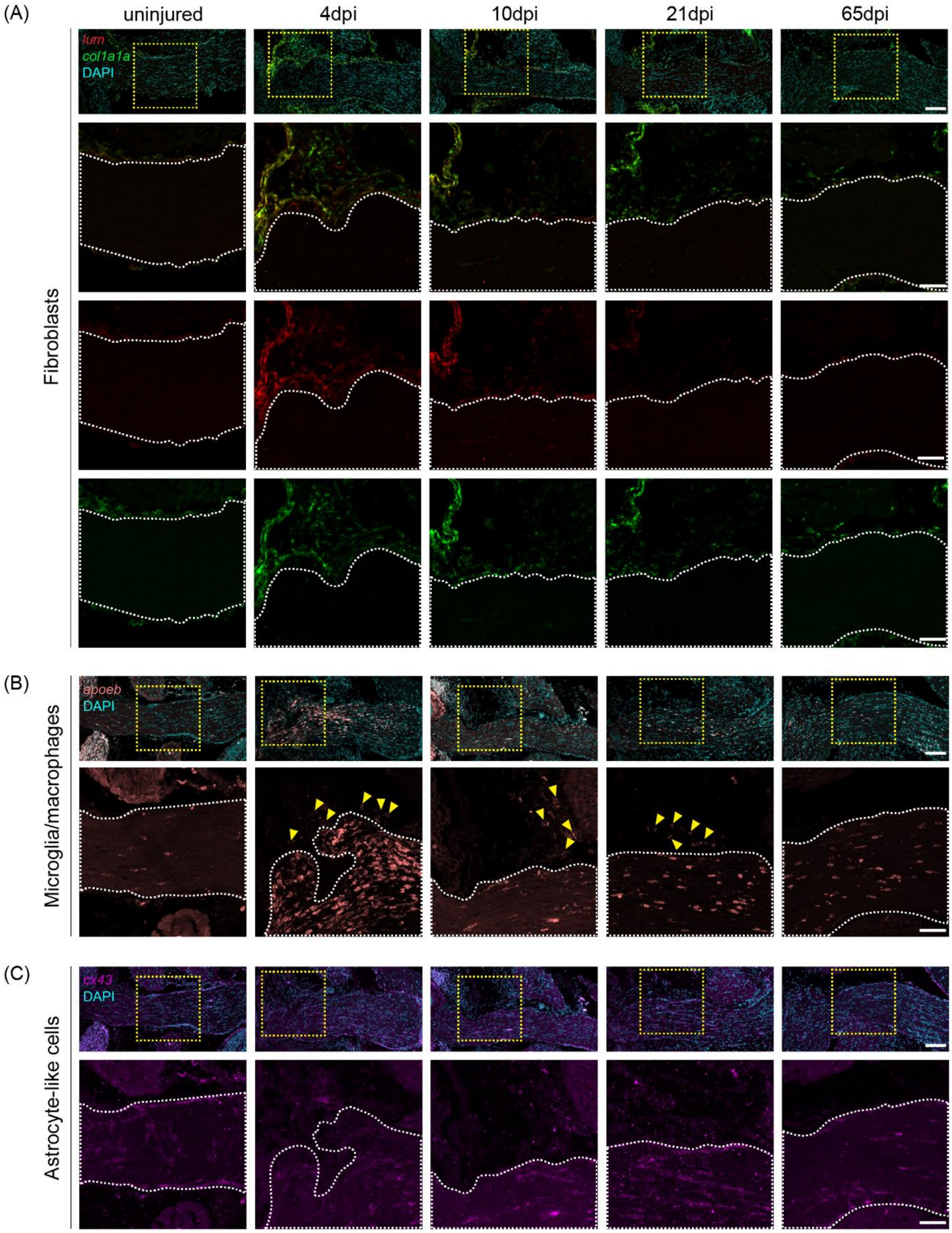

Supplementary figure S6: Transient fibrotic, immune and glial response after pONT.

(**A-D**) Representative HCR micrographs of complete optic nerve sections from young adult (6-week-old) killifish subjected to pONT, visualizing different cell types (N= 6 per condition). The yellow dotted outline marks the region shown magnified below, with the optic nerve border indicated by a white dotted line (N = 6 per condition). Scale bar optic nerve overview = 100 µm, scale bar zoom boxes = 50 µm.

(**A**) Fibroblasts, labeled using probe pairs for *lum* (red) and *col1a1a* (green), are mildly enriched in the perineural region surrounding the optic nerve at 4 dpi, particularly near the injury site, before gradually returning to baseline levels. Both *lum*- and *col1a1a*-positive fibroblasts display this profile, although *lum*-positive cells are resolved more rapidly (21 dpi) compared to *col1a1a*-positive cells (65 dpi).

(**B**) Microglia/macrophages (pink), visualized using probe pairs for *apoeb*, transiently accumulate at the injury site within the optic nerve at 4 dpi, with a small portion of cells expanding to the lesion site outside the nerve (yellow arrowheads). Over time, the number of immune cells in and around the nerve decreases, approaching near baseline levels by 65 dpi.

(**C**) Astrocyte-like cells (magenta), visualized using probe pairs for *cx43*, are mildly enriched from 4 dpi onward, before returning to uninjured baseline levels by 65 dpi. Astrocyte-like cells remain confined to the optic nerve. Of note, mild non-specific background staining is present but remains clearly distinguishable from the astrocyte-like morphology of the *cx43*-positive cells.

Abbreviations: *apoeb*, apolipoprotein Eb; *col1a1a*, collagen type 1 alpha 1a; *cx43*, connexin 43; DAPI, 4',6-diamidino-2-phenylindole; dpi, days post injury; *lum*, lumican; pONT, partial optic nerve transection.
